# Cross-Sectional Reference Data From 29 European Countries for 6 Frequently Used Depression Measures

**DOI:** 10.1001/jamanetworkopen.2025.17394

**Published:** 2025-06-25

**Authors:** Leili Riazy, Mia Grote, Gregor Liegl, Matthias Rose, Felix Fischer

**Affiliations:** 1 Charité-Universitätsmedizin Berlin, corporate member of Freie Universität Berlin and Humboldt Universität zu Berlin, Medizinische Klinik mit Schwerpunkt für Psychosomatik, Center for Patient-Centered Outcomes Research, Berlin, Germany; 2 Deutsches Zentrum für Psychische Gesundheit (DZPG), Berlin, Germany

## Abstract

**Importance:**

Cross-sectional reference values are needed to enable adequate interpretation of individual and group-level scores of frequently used depression measures in research and clinical practice.

**Objective:**

To provide age-, sex- and country-specific reference values for the PROMIS depression metric as well as 6 frequently used depression measures.

**Design, Setting, and Participants:**

Within the cross-sectional European Health Interview Survey (EHIS) wave 3, depressive symptoms were assessed using Patient Health Questionnaire (PHQ)-8 in a general European population from 2018 to 2020. PHQ-8 data were used to score depression severity on the Patient Reported Outcomes Measurement Information System (PROMIS) depression T-score metric and estimated reference scores for 6 other measures. EHIS wave 3 had response rates ranging from 12% to 78% with 22 countries having a response rate of over 50%. PHQ-8 responses from the general population across 29 European countries were analyzed from March to October 2023.

**Exposures:**

Completion of EHIS.

**Main Outcome and Measure:**

PROMIS depression T-score estimates.

**Results:**

A total of 287 530 participants from the general population (156 655 [54.1%] were female) provided PHQ-8 responses. The median (IQR) PROMIS depression T-score of the overall European population was 45.33 (39.11-51.68), 44.11 (38.06-50.40) for male participants, and 46.37 (40.00-52.76) for female participants. Lowest scores were observed in Serbia with a median (IQR) of 40.81 (35.45-46.59) and highest were observed in Iceland (48.60 [42.60-54.45]).

**Conclusions and Relevance:**

In this cross-sectional study of depressive symptom scores in the general population, scores differed by age, sex, and country. Item response theory modeling and quantile regression enabled modeling of depression questionnaire reference data on a common, instrument-independent metric.

## Key Points

**Question:**

How can self-reported depression reference data from the general population across 29 European countries become more accessible?

**Findings:**

The cross-sectional European Health Interview Survey (EHIS) wave 3 assessed depressive symptoms using the Patient Health Questionnaire-8 in 287 530 participants from the general population across 29 European countries. Patient-Reported Outcomes Measurement Information System (PROMIS) depression metric linking was used to establish reference scores for PROMIS, as well as 6 frequently used depression scales.

**Meaning:**

These findings suggest the PROMIS depression score can be used to interpret European values of self-reported depressive symptoms on frequently used depression scales.

## Teaser

This cross-sectional study evaluated age-, sex- and country-specific reference values for the Patient-Reported Outcomes Measurement Information System depression metric as well as 6 frequently used depression measures.

## Introduction

Depressive disorders are a leading contributor to disease burden in all regions of the world. The lifetime risk of a major depressive episode is estimated to be 14.6% in high- to middle-income countries,^1^ and depressive disorders were the second leading cause of years lived with disability worldwide in both 1990 and 2019,^2^ making them a priority in clinical and epidemiological research. A key factor for individual treatment success as well as public health policymaking is close monitoring of depressive symptoms on the individual and population level.^3^

A common method to assess the frequency and severity of depressive symptoms in clinical practice and research is the use of self-report questionnaires, so called patient-reported outcome measures (PROMs), in which respondents rate multiple items reflecting their experience of symptoms of depressive disorders. PROMs have been developed for different purposes, such as measuring severity of depressive symptoms (eg, Beck Depression Inventory II [BDI II]^4^), depression screening in primary care (eg, Patient-Health Questionnaire 9 [PHQ-9]^5^), assessment of depressive symptoms in epidemiological studies (eg, Center for Epidemiologic Studies Depression Scale [CES-D]^6^), measuring general psychological distress (eg, 6-item Kessler scale [K6]^7^), or to cater to specific situations in patient lives (eg, the Edinburgh Postnatal Depression Scale [EPDS]^8^ or Hospital Anxiety and Depression Scale [HADS]^9^). Hence, these measures differ in their content, length, and quality.^10^

This large number of available PROMs is a challenge for research into depression. A recent review investigated the use of 388 different PROMs in 450 depression trials and concluded that “heterogeneity in outcomes is a known source of research waste that prevents the comparability and combination of RCT results in meta-analyses.”^11^ To overcome these challenges and build more reliable evidence, a standardized and comparable assessment of depression across studies is desirable.^12,13^

One particularly promising approach to achieve such standardization is the use of modern psychometric frameworks such as item response theory (IRT) to model the relationship between item responses from different PROMs.^14^ Standardized IRT-based metrics have been developed for measures of depression,^15,16^ in particular, the Patient Reported Outcomes Measurement Information System (PROMIS) T-score metric.^17,18^ This metric allows estimation of severity of depression on a standardized scale with a mean (SD) of 50 (10) in the US general population, even when different PROMs are used. Common information across depression measures can be extracted, enhancing data comparability.

For most PROMs, a pressing challenge is the availability of general population reference data. Such reference data allow comparisons of individual or group level scores to those expected in the general population. General population reference data are commonly collected using specific depression measures in internet panels, where self-selection or response bias can be a threat to validity, particularly when concerned with obtaining representative data.^19^ Furthermore, reference values of a specific depression measure cannot be readily translated into other measures.

The European Health Interview Survey (EHIS) is a large-scale, cross-sectional study on health status, health care use, and health determinants across 32 European states. Given the sophisticated sampling scheme and data collection, these data can serve as an excellent source of general population reference scores.^20^ In its recent wave 3, participants from 29 countries completed the mental health assessment of the EHIS, using the PHQ-8 to assess depressive symptoms. With IRT modeling, these data can be transformed onto the standardized PROMIS depression metric, allowing for the calculation of expected scores for other PROMs. This highlights the utility of IRT methods to make depression data more comparable independently of the specific measure used.

The objective of the present study is to provide depression reference scores from the general population across Europe for the most widely used depression scales. We focus on country, age, and gender as stratification variables, as they are the most commonly reported and widely used demographic indicators and are easily available in clinical and research settings. We used results from previous IRT modeling studies^16^ to standardize PHQ-8 depression scores on the PROMIS T-score metric. We estimated specific depression scores for country, age, and sex, focusing on key quantiles. The resulting PROMIS scores are translated to the scales of commonly used depression measures, BDI-II, CES-D, EPDS, HADS, K6, and PHQ-2, PHQ-8, or PHQ-9.

## Methods

The EHIS is a large scale, cross-sectional survey conducted in the general population of European countries.^20^ Ethical approval and the need for informed consent were not required for this research due to the public and anonymous nature of the data we used. Data collection is regulated by Commission Implementing Regulation (EU) 2018/255, and access to anonymized microdata is governed by Regulation (EC) No. 223/2009 and Eurostat’s procedures for access to confidential data for scientific purposes. This report follows the Strengthening the Reporting of Observational Studies in Epidemiology (STROBE) reporting guideline for cross-sectional studies.

### Sample

In its third wave between 2018 and 2020, 303 505 respondents from all countries in the European Union except for Spain as well as from Norway, Iceland, and Serbia were included. A flowchart of all data exclusion steps is shown in eFigure 1 in Supplement 1. The aim of the EHIS survey was to provide comparable data on the health status, health determinants, and use of health services, as well as barriers to access to health services among EU citizens. The survey targeted individuals aged 15 years and over living in private households in the country’s territory. Eurostat required data collection in individual countries to be based on representative probability samples. These samples have been collected from a nationally installed sampling frame, which selects individuals or households at random. Different data collection modes have been permitted, ranging from self-administered, over the internet or telephone-based interviews, to face-to-face interviews. Personal (face-to-face) interviews have been regarded as the preferred mode of data collection. The study design and principles are extensively described in the methodological manual.^20,21^

There were differences in survey methodology across countries. The most notable differences occurred in the data collection modes, participation of proxy interviews, and the data collection period. The nonrespondent rate ranged from 12% to 78%, with 22 countries reaching a response rate of over 50%. High nonresponse rates coincided with the prohibition of proxy interviews in 6 of the 7 mentioned countries.^21^

### Measures

The PHQ-9 is a frequently used depression self-report questionnaire.^5^ It is based on the diagnostic criteria of depressive disorders of the *Diagnostic and Statistical Manual of Mental Disorders* (Fifth Edition) and contains 9 items, assessing the frequency of symptoms within the past 2 weeks. It was developed to facilitate screening for depressive disorders in primary care but also serves as a dimensional measure of severity of depression.

Depressive symptom severity in the EHIS was assessed using the PHQ-8, which omits 1 item about self-harm and suicidality.^20^ The sum score of the PHQ-8 ranges from 0 to 24. Linguistic and measurement equivalence of the PHQ-8 was established in the EHIS.^21,22^ PHQ-8 summary statistics are given in eTable 1 and frequencies of sum score groups in eTable 2 in Supplement 1. Amounts of partially or completely missing PHQ-8 replies per country are given in eTables 3 and 4 in Supplement 1.

The PROMIS emotional distress (ED)-depression is a standardized, instrument-independent T-score metric based on a graded response model, where the US general population has a mean (SD) score of 50 (10).^23^ Multiple legacy depression scales such as the BDI-II, CES-D, EPDS, HADS, K6, and PHQ-9 have previously been cocalibrated with the PROMIS depression metric (see eTable 5 in Supplement 1).^16,24,25^ Hence, these questionnaires can be scored on the PROMIS depression T-score metric and vice versa.

### Statistical Analysis

We report sociodemographic variables of the sample descriptively, with categorical variables in absolute and relative frequencies and continuous as mean (SD). Using individual responses to the PHQ-8 items and previously published IRT item parameters,^16^ we estimated PROMIS depression T-scores.^26^ In case of missing item responses, T-scores were estimated based on the available responses.^18,27^ No new IRT calibration was performed. For further analysis, we considered the true depression score as missing, with the observed scores and their standard errors indicating a normal likelihood distribution of the true scores. For each observed score, we imputed 25 plausible true score values from that likelihood distribution.^18,27^ Statistical analysis was conducted in each of these 25 imputed datasets and results were pooled following Rubin rule.

To model the association between sociodemographic variables (age, sex, and country) and PROMIS T-scores, a series of explanatory regression models were fitted. These models were increasingly complex by including interaction terms between confounding variables as well as modeling age using an interpolated spline. To model the entire distribution of depression data, quantile regression models were fitted to model the dependence of the sociodemographic variables on different quantiles of PROMIS T-scores. Model selection was performed using bayesian information criterion (BIC). A detailed description of the different explanatory formulas and the selection process is given in eAppendix 2 in Supplement 1.

We then estimated the expected PROMIS T-scores at the 25th, 50th, 75th, 90th, 95th, and 99th percentiles given the confounding variables of age, sex, country, and their respective interactions. The estimated PROMIS T-scores were then transformed to the scales of the BDI II, CES-D, EPDS, HADS, K6, and PHQ-2, PHQ-8, or PHQ-9.^16,24,25^ Statistical analysis was performed using R version 4.5.0 (R Project for Statistical Computing), in particular the packages mirt and quantreg.

## Results

From 303 505 samples in the microdata, 14 065 did not provide any PHQ-8 data and 287 530 participants (156 655 [54.1%] female) were included for further analysis. The percentage of completely missing PHQ-8 assessments was lower than 5% in 25 countries. Information on missing data by country and a detailed flowchart is provided in eFigure 1 in Supplement 1. Descriptions for age, sex, urbanization, income, job status, and marital status are provided in Table 1 and details for each country in eAppendix 1 in Supplement 1.

**Table 1.** Participant Demographics

Variable	Participants, No. (%)	
	Unweighted	Weighted
Age, y		
15-19	14 276 (4.9)	19 869 123 (6.1)
20-24	13 874 (4.8)	19 261 332 (5.9)
25-29	15 257 (5.3)	21 830 415 (6.7)
30-34	17 630 (6.1)	25 106 796 (7.7)
35-39	19 865 (6.9)	25 513 761 (7.8)
40-44	22 205 (7.7)	26 839 721 (8.2)
45-49	23 324 (8.1)	26 892 842 (8.2)
50-54	25 195 (8.7)	29 126 581 (8.9)
55-59	25 953 (9.0)	27 097 496 (8.3)
60-64	26 739 (9.2)	26 203 456 (8.0)
65-69	26 294 (9.1)	23 383 258 (7.2)
70-74	22 197 (7.7)	19 466 432 (6.0)
≥75	36 631 (12.7)	35 919 969 (11.0)
Sex		
Female	156 655 (54.1)	170 357 058 (52.2)
Male	132 785 (45.9)	156 154 125 (47.8)
Urbanization		
Densely populated	94 507 (32.7)	116 533 543 (35.7)
Intermediately populated	91 225 (31.5)	109 348 450 (33.5)
Thinly populated	90 392 (31.2)	91 752 597 (28.1)
Missing	13 316 (4.6)	8 876 593 (2.7)
Income		
<Q1	49 644 (17.2)	58 692 131 (18.0)
Q1-<Q2	54 115 (18.7)	61 135 682 (18.7)
Q2-<Q3	56 143 (19.4)	62 547 051 (19.2)
Q3-<Q4	57 205 (19.8)	64 705 280 (19.8)
Q4-<Q5	55 806 (19.3)	63 211 121 (19.4)
Missing	16 527 (5.7)	16 219 917 (5.0)
Job status		
Employed	138 616 (47.9)	169 469 834 (51.9)
Unemployed	14 755 (5.1)	16 262 429 (5.0)
Retired	86 124 (29.8)	81 030 665 (24.8)
Unable to work due to long-standing health problems	5973 (2.1)	7 532 397 (2.3)
Student or pupil	21 017 (7.3)	27 189 622 (8.3)
Fulfilling domestic tasks	15 226 (5.3)	17 703 068 (5.4)
Compulsory military or civilian service	927 (0.3)	517 930 (0.2)
Other	5255 (1.8)	5 154 745 (1.6)
Missing	1547 (0.5)	1 650 494 (0.5)
Marital status		
Never married and never been in a registered partnership	79 564 (27.5)	105 704 463 (32.4)
Married or in a registered partnership	155 475 (53.7)	164 329 863 (50.3)
Widowed or in registered partnership that ended with death of partner	30 349 (10.5)	29 615 562 (9.1)
Divorced or in registered partnership that was legally dissolved	23 230 (8.0)	26 096 106 (8.0)
Missing	822 (0.3)	765 188 (0.2)

Abbrevation: Q, quintile.

Table 2 shows the PROMIS depression scores per country. Overall, we found a mean (SD) PROMIS depression score of 45.43 (9.84) across Europe. A notable floor effect, meaning many participants reporting no depressive symptoms, was observed, from 16.5% in Iceland to 67.3% in Serbia.

**Table 2.** Summary Statistics for PROMIS Depression T-Scores

Country	Mean (SD) score	Median (IQR) score	Patients, No.	Patients at score floor, %^a^
Austria	45.5 (7.6)	43.7 (37.5-50.2)	15 253	4794 (31.4)
Belgium	47.4 (8.7)	45.9 (37.6-52.8)	7790	1946 (25.0)
Bulgaria	43.5 (8.5)	37.5 (37.5-47.8)	7216	3900 (54.0)
Croatia	46.4 (8.9)	45.5 (37.5-52.2)	5225	1797 (34.4)
Cyprus	41.4 (7.2)	37.5 (37.5-42.7)	5906	4107 (69.5)
Czechia	44.3 (7.5)	42.7 (37.5-48.6)	7884	3254 (40.0)
Denmark	46.9 (9.1)	45.5 (37.5-52.6)	6422	1928 (30.0)
Estonia	48.8 (8.1)	48.0 (42.7-54.5)	4850	814 (16.8)
Finland	45.8 (8.3)	44.9 (37.5-51.8)	5846	2011 (34.4)
France	48.2 (8.3)	47.6 (42.7-53.6)	13 908	2727 (19.6)
Germany	46.5 (7.7)	45.5 (41.8-51.5)	22 996	5625 (24.5)
Greece	41.6 (7.0)	37.5 (37.5-43.7)	7847	5194 (66.2)
Hungary	46.3 (8.4)	45.5 (37.5-52.0)	5580	1775 (31.8)
Iceland	48.9 (8.3)	48.4 (42.7-54.2)	3870	639 (16.5)
Ireland	43.3 (8.1)	37.5 (37.5-46.9)	7574	3998 (52.8)
Italy	44.1 (7.9)	42.7 (37.5-48.7)	39 077	1735 (44.4)
Latvia	45.2 (8.0)	43.1 (37.5-50.2)	5846	2161 (37.0)
Lithuania	45.7 (8.7)	43.4 (37.5-51.8)	4826	1824 (37.8)
Luxembourg	48.6 (8.9)	47.8 (41.8-54.6)	4451	924 (20.8)
Malta	45.8 (7.7)	44.3 (37.5-51.4)	4347	1462 (33.6)
Netherlands	47.6 (8.2)	46.9 (41.8-52.8)	8193	1874 (22.9)
Norway	45.2 (7.8)	43.7 (37.5-50.1)	7905	2763 (35.0)
Poland	45.4 (8.2)	42.7 (37.5-51.1)	16 830	6201 (36.8)
Portugal	46.8 (9.4)	45.5 (37.5-53.3)	14 582	4977 (34.1)
Romania	45.0 (8.7)	42.7 (37.5-51.2)	15 889	7270 (45.8)
Serbia	41.2 (6.6)	37.5 (37.5-42.7)	12 456	8383 (67.3)
Slovakia	43.5 (7.6)	41.8 (37.5-47.6)	5526	2652 (48.0)
Slovenia	47.4 (8.6)	45.9 (41.8-53.0)	9745	2395 (24.6)
Sweden	47.1 (9.5)	45.5 (37.5-53.8)	9690	3108 (32.1)

^a^ The percentage of scores equal to zero in the PHQ-8 (floor) is shown.

Including age, sex, and country significantly improved the quantile regression, indicating that PROMIS depression scores vary by these factors. Spline-interpolated age further improved model fit. Among the various combinations of confounders, interactions between age and country demonstrated the lowest BIC values and were therefore used for further analysis. eAppendix 2 in Supplement 1 explains this finding in detail and all model fit parameters are given in eTable 6 in Supplement 1.

The 25th, 50th, 75th, 90th, 95th, and 99th percentiles for the overall European population are estimated to be 39.11, 45.33, 51.68, 57.53, 61.38, and 70.05, respectively. Overall, we observed highest median (IQR) PROMIS T-scores in Iceland (48.60 [42.60-54.45]), and lowest in Serbia (40.81 [35.45-46.59]).

**Figure 1.**
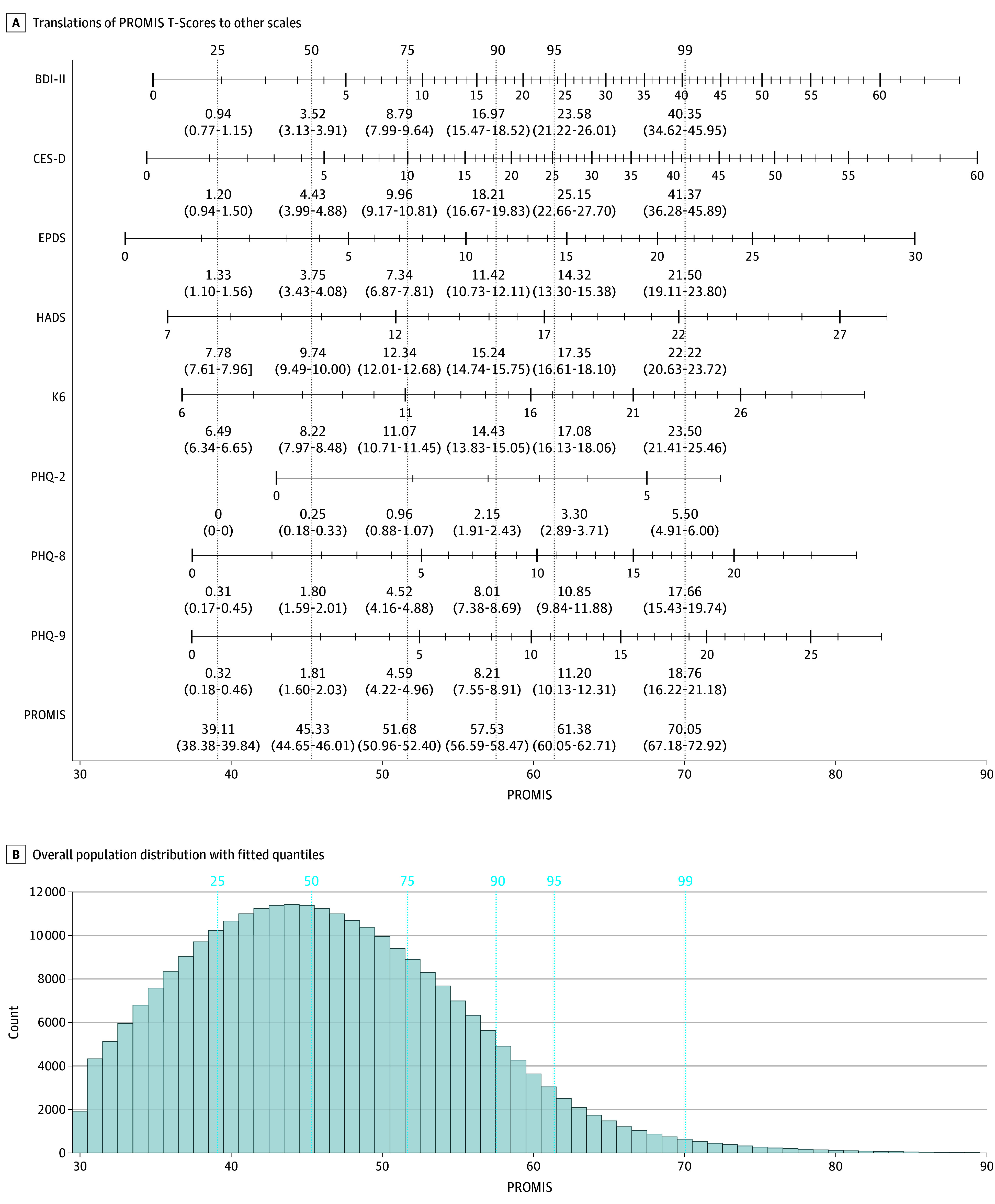
Patient-Reported Outcomes Measurement Information System (PROMIS) T-Score Quantiles Along With Corresponding Expected Questionnaire Sum Scores and Histogram of Estimated PROMIS Depression T-Score Values Above the x-axis for the PROMIS score value, multiple other axes of other questionnaires are arranged to show the corresponding sum scores respectively. To highlight the 25th, 50th, 75th, 90th, 95th, and 99th percentiles, vertical lines with corresponding questionnaire sum scores are included. BDI II indicates Beck Depression Inventory II; CES-D, Center for Epidemiologic Studies Depression Scale; EPDS, Edinburgh Postnatal Depression Scale; HADS, Hospital Anxiety and Depression Scale; K6, 6-item Kessler scale; PHQ, Patient-Health Questionnaire.

The median (IQR) T-score of the overall population of 45.33 (39.11-51.68) translates to the following questionnaire sum scores: BDI-II, 3.52; CES-D, 4.43; EPDS, 3.75; HADS, 9.74; K6, 8.22; PHQ-2, 0.25; PHQ-8, 1.80; and PHQ-9, 1.81. Figure 1 shows the overall distribution of PROMIS depression scores in Europe.

**Figure 2.**
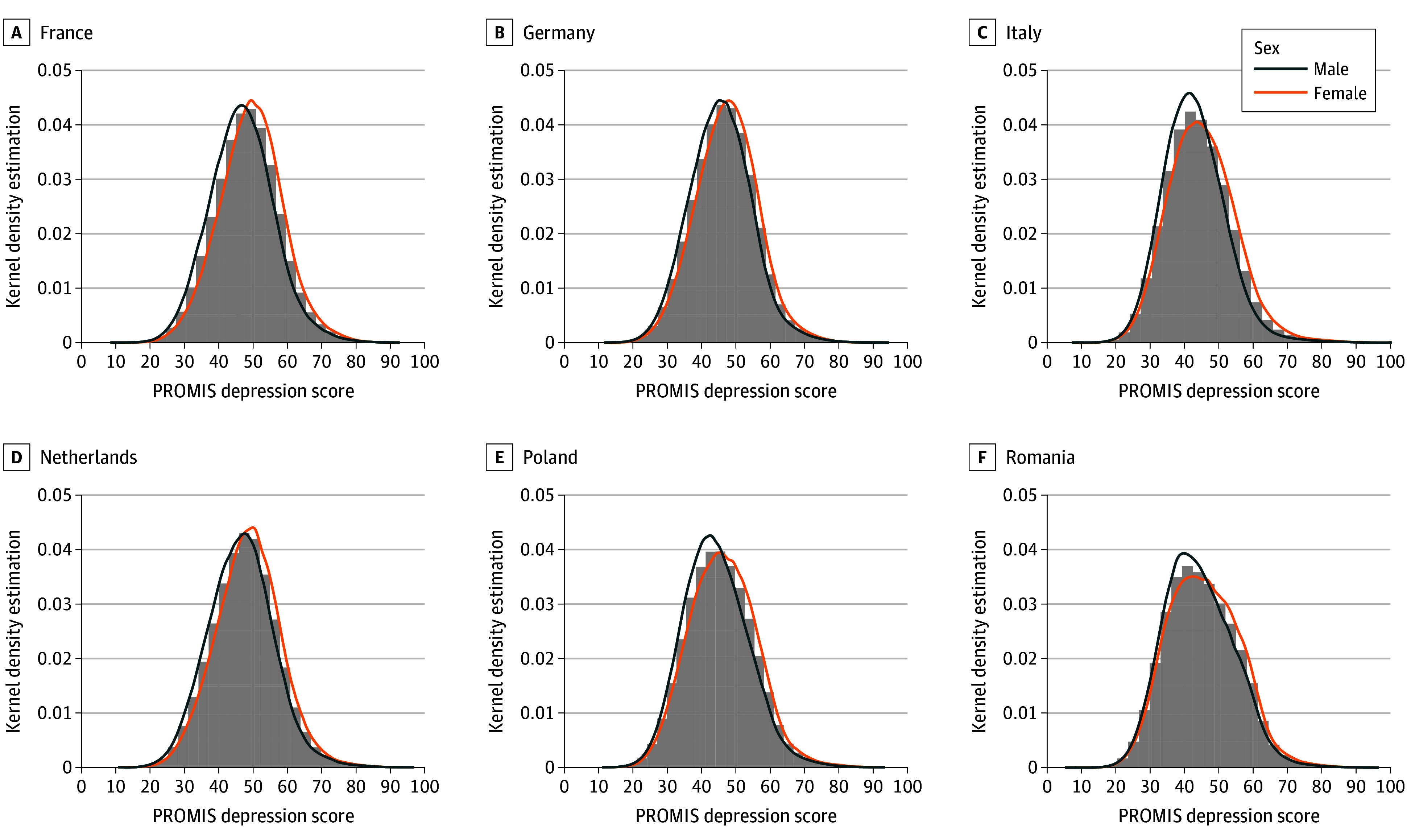
Patient-Reported Outcomes Measurement Information System (PROMIS) T-Score Kernel Density Estimations and Histograms per Country and Sex for the 6 Largest European Countries Included Data reported from the 6 included countries with the largest populations. For all other countries, see eFigure 2 in Supplement 1.

We found that independent from country and age group, women reported higher severity of depression than men. Overall, the median (IQR) PROMIS depression T-score in men was 44.11 (38.06-50.40) and in women was 46.37 (40.00-52.76). In general, the distribution of PROMIS scores in the female population is slightly higher than in the male population for all countries. For a more detailed view on specific countries in the collective, individual distributions of PROMIS scores per country and per sex are shown in Figure 2 and in detail in eFigure 2 in Supplement 1.

**Figure 3.**
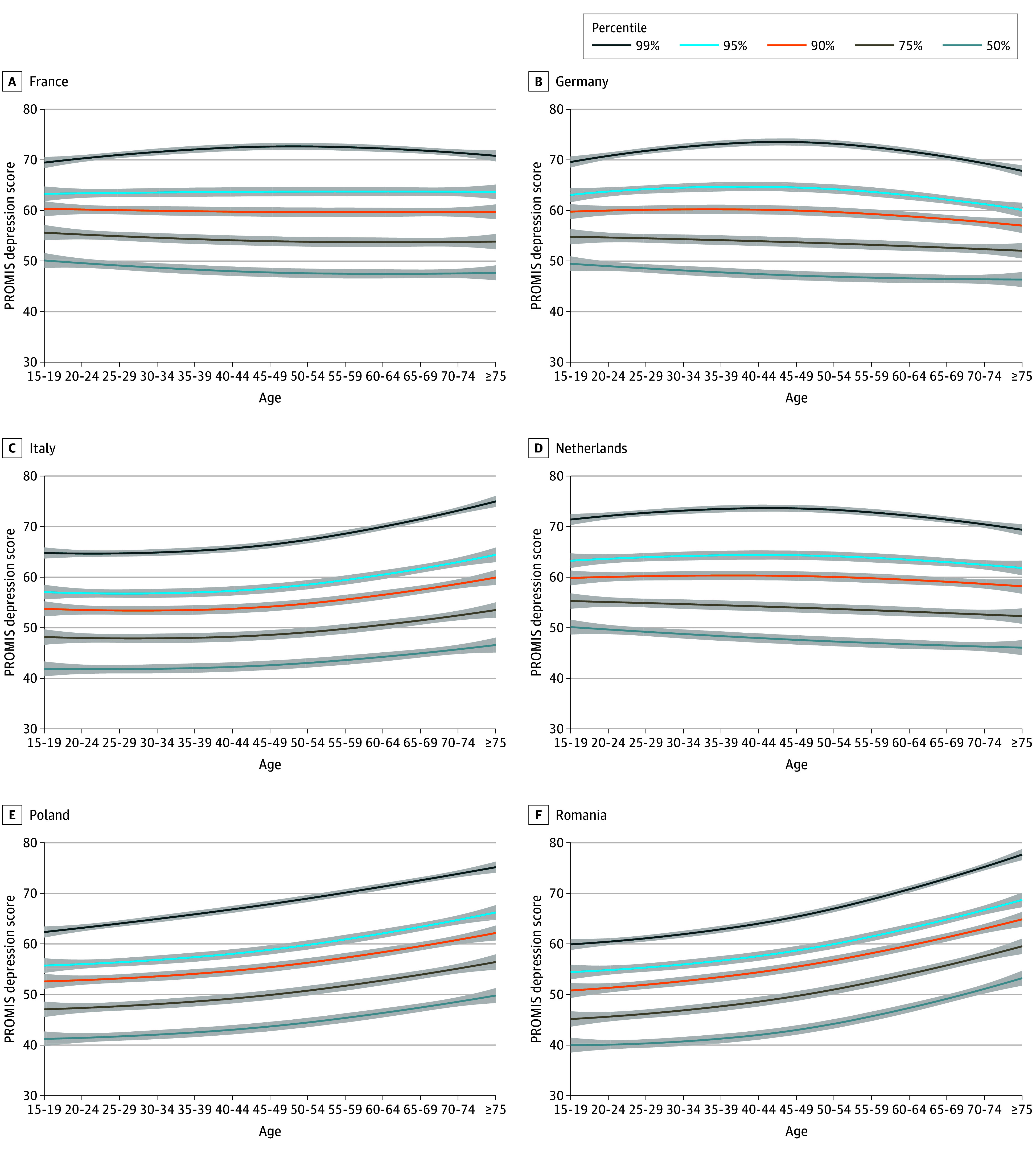
Patient-Reported Outcomes Measurement Information System (PROMIS) T-Score Line Plots per Age Group and 50th, 75th, 90th, 95th, and 99th Percentiles for the 6 Largest European Countries Included Data reported from the 6 included countries with the largest population.

Depression scores varied considerably between different age groups and the pattern was different across countries. For example, Sweden and Norway show a decreasing PROMIS depression score with increasing age, whereas increasing scores were observed in Romania, Serbia, and Bulgaria. Some countries showed a pattern where highest scores were observed in medium age groups. These patterns were most pronounced for respondents with high severity of depression in the 90th, 95th, and 99th percentiles. The expected scores for the respective percentiles across age and country are presented in Figure 3 and in more detail in eFigure 3 in Supplement 1.

We provide a web application that shows reference values stratified by age, sex, and country.^28^ This allows fine-grained comparisons of both individual and group-level depression data to the expected scores derived from general population samples.

## Discussion

In this cross-sectional study, we report population-based reference values for the PROMIS depression metric across 29 European countries. We found that age, sex, and country as well as their interactions are associated with the expected depression scores in the general population. Use of modern psychometric models enabled us to translate reference scores to an instrument-independent common metric as well as to 6 of the most relevant depression measures, facilitating comparability of study results and individual patient data to the European general population.

Our study significantly extends the availability of general population reference data for depression self-report scales across Europe. A systematic literature search for such data limited to the past decade resulted in only 5 studies^29,30,31,32,33^ reporting general population data on severity of depression. These studies were limited to single countries (Belgium, Hungary, Netherlands, Germany, and Norway), used different PROMs (CESD, PHQ-9, and PROMIS Short Form v1.0-depression 4a, 6a, 8a, and 8b) and tend to have smaller sample sizes per country (837 to 6165). In addition, the studies differed in their respective sampling schemes, inclusion criteria, and methodological approaches, making it difficult to compare the reference data provided. In contrast, the reference values we derived from EHIS are based on a broad, population-based study with comparable design in each country, ensuring a high-degree of validity of cross-country comparisons.^34^ Furthermore, the scaling of PHQ-8 data on the construct-based PROMIS depression scale enables interpretation of this reference data for the most frequently used depression measures in epidemiological research and clinical practice.

Overall, we found a mean (SD) PROMIS depression score of 45.43 (9.84) across Europe. Given that PROMIS T-scores have been scaled to a mean (SD) of 50 (10) in the US general population,^23^ scores and their variability are lower than expected. With our study design, we cannot tell whether this was due to differences in sampling schemes, systematic and/or random errors in the score calibration, or whether this reflects the actual difference in self-reported depression between the US and Europe. In a previous study^18^ in 461 patients with multiple sclerosis, the PROMIS scores derived by PHQ items and the PROMIS short form were found to deviate by 3 T-scores. There, the PHQ items were overestimating the PROMIS depression score. Furthermore, we cannot rule out that expected scores on legacy depression measures are biased, in particular as similar models^15^ would yield different expected scores. Despite such potential biases in prediction of expected scores, the reference data reported here provides valuable information for score interpretation in studies and individual assessment, for example when monitoring depressive symptoms during treatment in clinical practice.^34^

So far, analysis of the EHIS depression data focused on the proportion of participants achieving scores equal to and greater than 10.^34,35^ This dichotomization overestimates true depression prevalence considerably.^36,37^ and there is no evidence that the optimal cutoff for screening^38^ is a meaningful classifier for depression severity (eg, meaningful vs not meaningful symptom burden). At best, such an approach leads to a loss of information, reducing statistical power and obscuring nuanced relationships within the data; at worst, it is misleading and harmful for health policymaking.^36^ Our analysis shows how dimensional assessment of depression severity yields additional insights compared with categorical treatment. For example, the association between age and depression score is more extreme in those with the highest scores (Figure 3). This might have important implications for public health policymaking, for example in identifying vulnerable groups. Instead of focusing on the prevalence of certain conditions, Rose et al^39^ have proposed to reduce the disease burden by improving health on a population level, in particular as mental health exists on a continuum.^40^

### Limitations

Several limitations need to be considered when using the reference data presented. First, selection bias cannot be ruled out in the EHIS since people with certain characteristics (eg, old age or high severity of depression) might be less likely to respond to the survey. Other sources of bias could be caused by varying data collection modes across countries and varying response rates. The PHQ-8 showed cross-country equivalence in the second EHIS wave.^22^

A second limitation is that the IRT model used to translate questionnaire scores to the PROMIS T-score metric was estimated previously on different datasets (listed in eTable 5 in Supplement 1), which might not accurately represent the current conditions. Furthermore, accounting for imprecision of previously estimated item parameters in score estimation was not feasible given model complexity and the large sample size.

Therefore, researchers and clinicians should take into account potential imprecision and bias of linked scores and interpret the values with appropriate caution. More extensive validation of the underlying IRT model across different populations is desirable.

Since we have only used sex, age, and country as variables for depression scores, our model cannot account for differential severity of depressive symptoms in specific sociodemographic groups. The reference values therefore need to be interpreted with caution in populations with high or low income or who are unable to work due to long-standing health problems.

## Conclusions

We have shown that self-reported depression severity varies with age, sex, and country in the general population in Europe, and we used quantile regression to model these associations across the entire range of depression severity, providing a more detailed perspective than mere modeling of mean scores or scores above an arbitrary cutoff. Furthermore, modeling the EHIS data on an instrument-independent, construct-based common metric allow these data to be used as a reference for the most widely used depression scales. It is therefore an important step toward the establishment of a comparable, standardized scale for self-reported depression severity.

## Notes

Supplement 1. **eFigure 1.** Flowchart of All Data Exclusion Steps **eTable 1.** PHQ-8 Summary Statistics **eTable 2.** PHQ-8 Frequency Report **eTable 3.** Missing PHQ-8 Data Including Proxy Interviews **eTable 4.** Missing PHQ-8 Data Excluding Proxy Interviews **eTable 5.** Demographic Information on Samples Used in Applied Linking Study **eAppendix 1.** Country-Specific Demographics Tables **eAppendix 2.** Model Selection for Quantile Regression **eTable 6.** Parameters From Fitted Quantile Regression Model **eFigure 2.** PROMIS T-Score Distributions for 29 European Countries **eFigure 3.** Quantile Regression Fit Quality Control

Supplement 2.
Data Sharing Statement
