# Supplementary material for: Cross-Sectional Reference Data From 29 European Countries for 6 Frequently Used Depression Measures
Source: JAMA Netw Open. 2025 Jun 25;8(6):e2517394. doi: 10.1001/jamanetworkopen.2025.17394 (PMC12199077; doi:10.1001/jamanetworkopen.2025.17394)
Supplement: Supplement 1. — eFigure 1. Flowchart of All Data Exclusion Steps eTable 1. PHQ-8 Summary Statistics eTable 2. PHQ-8 Frequency Report eTable 3. Missing PHQ-8 Data Including Proxy Interviews eTable 4. Missing PHQ-8 Data Excluding Proxy Interviews eTable 5. Demographic Information on Samples Used in Applied Linking Study eAppendix 1. Country-Specific Demographics Tables eAppendix 2. Model Selection for Quantile Regression eTable 6. Parameters From Fitted Quantile Regression Model eFigure 2. PROMIS T-Score Distributions for 29 European Countries eFigure 3. Quantile Regression Fit Quality Control [file jamanetwopen-e2517394-s001.pdf]

Supplemental Online Content

Riazy L, Grote M, Liegl G, Rose M, Fischer F. Cross-sectional reference data from 29 European countries for 6 frequently used depression metrics. *JAMA Netw Open*. 2025;8(6):e2517394. doi:10.1001/jamanetworkopen.2025.17394

eFigure 1. Flowchart of All Data Exclusion Steps
eTable 1. PHQ-8 Summary Statistics
eTable 2. PHQ-8 Frequency Report
eTable 3. Missing PHQ-8 Data Including Proxy Interviews
eTable 4. Missing PHQ-8 Data Excluding Proxy Interviews
eTable 5. Demographic Information on Samples Used in Applied Linking Study
eAppendix 1. Country-Specific Demographics Tables
eAppendix 2. Model Selection for Quantile Regression
eTable 6. Parameters From Fitted Quantile Regression Model
eFigure 2. PROMIS T-Score Distributions for 29 European Countries
eFigure 3. Quantile Regression Fit Quality Control

This supplemental material has been provided by the authors to give readers additional information about their work.

© 2025 Riazy L et al. *JAMA Network Open*.

eFigure 1. Flow-chart of All Data Exclusion Steps

The following flow-chart shows all steps of data exclusion that were done during our analysis. The top box represents the microdata that we received. The box on the bottom right represents the final dataset that went into the analysis.

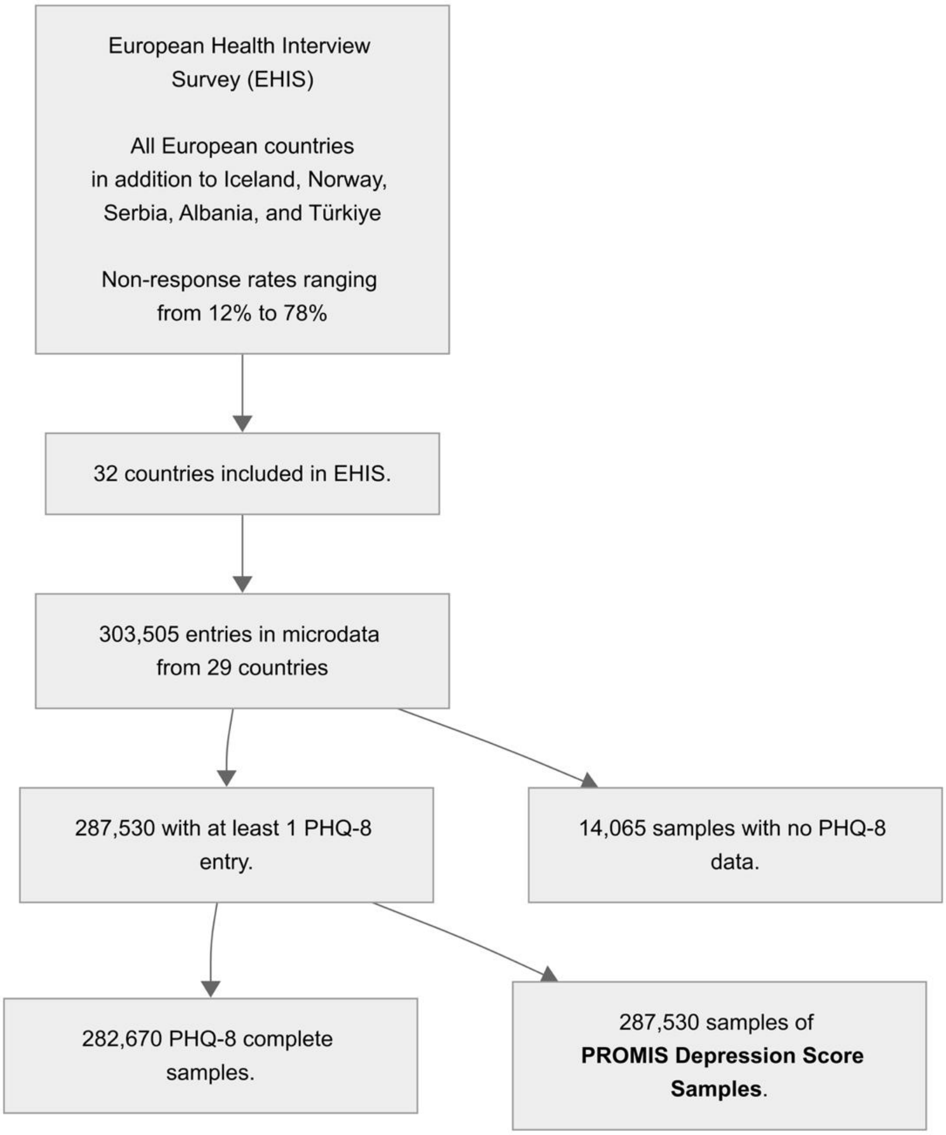

© 2025 Riazy L et al. *JAMA Network Open*.

eTable 1. PHQ-8 Summary Statistics

The following table summarizes information on the PHQ-8 sum score for 29 European countries. SD = standard deviation, IQR = interquartile range, floor = proportion of samples equal to zero.

Country	mean	SD	median	IQR	n	floor
Cyprus	1.2	2.9	0	1	5,906	69.5%
Serbia	1.2	2.7	0	1	12,463	67.3%
Greece	1.2	2.7	0	1	7,848	66.2%
Bulgaria	2.0	3.7	0	2	7,231	53.9%
Ireland	2.0	3.6	0	2	7,621	52.5%
Slovakia	2.0	3.2	1	3	5,527	48.0%
Romania	2.4	3.6	1	3	15,908	45.7%
Italy	2.2	3.5	1	3	39,368	44.1%
Czechia	2.2	3.2	1	3	7,884	40.0%
Lithuania	2.6	3.7	1	4	4,836	37.7%
Latvia	2.5	3.5	1	4	5,851	36.9%
Poland	2.5	3.5	1	4	16,865	36.8%
Norway	2.5	3.4	1	4	7,913	34.9%
Croatia	3.1	4.1	2	4	5,230	34.4%
Finland	2.7	3.5	1	4	5,911	34.0%
Portugal	3.3	4.3	2	5	14,617	34.0%
Malta	2.8	3.2	2	4	4,356	33.6%
Sweden	3.4	4.5	2	5	9,757	31.9%
Hungary	2.8	3.7	2	4	5,603	31.7%
Austria	2.6	3.3	2	4	15,253	31.4%
Denmark	3.2	4.3	2	4	6,629	29.1%
Germany	3.0	3.5	2	3	23,001	24.5%
Slovenia	3.4	4.0	2	5	9,763	24.5%
Netherlands	3.6	3.9	2	4	8,194	22.9%
Belgium	3.5	4.2	2	5	8,761	22.2%
Luxembourg	3.8	4.3	2	4	4,504	20.5%
France	3.9	4.1	3	5	13,908	19.6%
Estonia	3.9	3.9	3	5	4,851	16.8%
Iceland	4.0	4.1	3	5	3,881	16.5%

© 2025 Riazy L et al. *JAMA Network Open*.

eTable 2. PHQ-8 Frequency Report

The following table shows the count of PHQ-8 sum score per bin and country. The countries are named by their international country codes.

PHQ-8 Score	AT	BE	BG	CY	CZ	DE	DK	EE	EL	FI
[0,5]	13,224	5,915	6,248	5,485	6,923	18,751	4,985	3,585	7,283	4,688
(5,10]	1,609	1,023	490	296	729	2,786	748	903	358	650
(10,15]	466	370	196	69	167	740	254	247	92	148
(15,24]	162	185	111	56	63	273	182	101	50	78
	<20	2,151	495	250	111	451	460	45	342	687

PHQ-8 Score	FR	HR	HU	IE	IS	IT	LT	LU	LV	MT
[0,5]	10,446	4,160	4,495	6,638	2,770	39,206	3,861	3,136	5,024	3,646
(5,10]	2,629	664	742	565	658	3,631	486	657	624	576
(10,15]	786	236	147	177	194	1,119	120	197	174	89
(15,24]	331	116	74	129	101	818	72	119	72	20-49
	<20	285	145	112	158	1,188	384	395	139	69

PHQ-8 Score	NL	NO	PL	PT	RO	RS	SE	SI	SK
[0,5]	6,296	6,689	14,141	11,064	13,234	11,572	7,138	7,480	4,919
(5,10]	1,338	822	1,775	2,038	1,786	619	1,234	1,399	440
(10,15]	347	223	425	624	355	131	448	378	107
(15,24]	163	92	216	398	258	84	304	203	54
	50	87	3,402	493	553	772	633	440	<20

© 2025 Riazy L et al. *JAMA Network Open*.

eTable 3. Missing PHQ-8 Data Including Proxy Interviews

The following table shows the percentage of missing PHQ-8 replies in the microdata for 29 European countries. The columns differentiate whether the PHQ-8 replies are only partially or completely missing.

Country	Not complete PHQ-8 data: count (relative)	No PHQ-8 data: count (relative)
Austria	0 (0.0%)	0 (0.0%)
Belgium	2151 (22.3%)	1854 (19.2%)
Bulgaria	495 (6.6%)	324 (4.3%)
Croatia	285 (5.2%)	236 (4.3%)
Cyprus	250 (4.1%)	250 (4.1%)
Czechia	111 (1.4%)	109 (1.4%)
Denmark	460 (6.9%)	207 (3.1%)
Estonia	45 (0.9%)	31 (0.6%)
Finland	687 (11.0%)	405 (6.5%)
France	0 (0.0%)	0 (0.0%)
Germany	451 (2.0%)	5 (0.0%)
Greece	342 (4.2%)	278 (3.4%)
Hungary	145 (2.6%)	23 (0.4%)
Iceland	158 (4.1%)	11 (0.3%)
Ireland	112 (1.5%)	47 (0.6%)
Italy	1188 (2.6%)	1188 (2.6%)
Latvia	139 (2.3%)	96 (1.6%)
Lithuania	384 (7.8%)	97 (2.0%)
Luxembourg	395 (8.8%)	53 (1.2%)
Malta	69 (1.6%)	66 (1.5%)
Netherlands	50 (0.6%)	1 (0.0%)
Norway	87 (1.1%)	8 (0.1%)
Poland	3402 (17.0%)	3129 (15.7%)
Portugal	493 (3.4%)	35 (0.2%)
Romania	553 (3.4%)	24 (0.1%)
Serbia	772 (5.9%)	722 (5.5%)
Slovakia	7 (0.1%)	1 (0.0%)
Slovenia	440 (4.4%)	155 (1.6%)

© 2025 Riazy L et al. *JAMA Network Open*.

Country	Not complete PHQ-8 data: count (relative)	No PHQ-8 data: count (relative)
Sweden	633 (6.5%)	67 (0.7%)

© 2025 Riazy L et al. *JAMA Network Open*.

eTable 4. Missing PHQ-8 Data— Excluding Proxy Interviews

The following table shows the percentage of missing PHQ-8 replies in the microdata for 29 European countries. All proxy interviews were excluded from the dataset prior to counting. The columns differentiate whether the PHQ-8 replies are only partially or completely missing.

Country	Not complete PHQ-8 data: count (relative)	No PHQ-8 data: count (relative)
Austria	0 (0.0%)	0 (0.0%)
Belgium	1268 (14.5%)	971 (11.1%)
Bulgaria	186 (2.6%)	15 (0.2%)
Croatia	54 (1.0%)	5 (0.1%)
Cyprus	0 (0.0%)	0 (0.0%)
Czechia	2 (0.0%)	0 (0.0%)
Denmark	460 (6.9%)	207 (3.1%)
Estonia	15 (0.3%)	1 (0.0%)
Finland	347 (5.9%)	65 (1.1%)
France	0 (0.0%)	0 (0.0%)
Germany	451 (2.0%)	5 (0.0%)
Greece	65 (0.8%)	1 (0.0%)
Hungary	145 (2.6%)	23 (0.4%)
Iceland	158 (4.1%)	11 (0.3%)
Ireland	112 (1.5%)	47 (0.6%)
Italy	291 (0.7%)	291 (0.7%)
Latvia	43 (0.7%)	5 (0.1%)
Lithuania	297 (6.1%)	10 (0.2%)
Luxembourg	395 (8.8%)	53 (1.2%)
Malta	12 (0.3%)	9 (0.2%)
Netherlands	50 (0.6%)	1 (0.0%)
Norway	87 (1.1%)	8 (0.1%)
Poland	308 (1.8%)	35 (0.2%)
Portugal	493 (3.4%)	35 (0.2%)
Romania	531 (3.3%)	19 (0.1%)
Serbia	57 (0.5%)	7 (0.1%)
Slovakia	7 (0.1%)	1 (0.0%)

© 2025 Riazy L et al. *JAMA Network Open*.

Country	Not complete PHQ-8 data: count (relative)	No PHQ-8 data: count (relative)
Slovenia	303 (3.1%)	18 (0.2%)
Sweden	633 (6.5%)	67 (0.7%)

© 2025 Riazy L et al. *JAMA Network Open*.

eTable 5. Demographic Information on Samples Used in Applied Linking Study

In our study, several transformations were used for translating questionnaire replies. These transformations were previously established in so-called linking studies.

In the following, we present a table summarizing basic demographic information alongside the measures: PROMIS Depression: Patient-Reported Outcomes Measurement Information System – Depression, CES-D: Center for Epidemiological Studies Depression Scale, PHQ-9: Patient Health Questionnaire-9, K6: Kessler Psychological Distress Scale, BDI-II: Beck Depression Inventory-II, HADS Depression: Hospital Anxiety and Depression Scale – Depression subscale, EPDS: Edinburgh Postnatal Depression Scale.

Measure	Sample Size	Source	Demographics	Depression Severity
PROMIS Depression[1]	14,839	Reference metric (NIH PROMIS initiative).	Matched to 2000 U.S. Census (gender, age, race, education).	T-score metric: 50 = U.S. general population mean; 1 SD = 10.
CES-D[2]	747	PROMIS Wave I (Polimetrix/YouGov panel). national, web-based polling firm	- 48.1% male<br>- 80.5% White<br>- Mean age: 51.3<br>- 77.2% with post-high school education.	24% scored ≥16 ("indicating moderate depression").
PHQ-9[2]	748	NIH Toolbox Study (Greenfield Online/Toluna panel).	- 43.9% male<br>- 80.1% White<br>- Mean age: 47.2<br>- 68.0% with post-high school education.	21% scored ≥10 ("indicating moderate depression").
K6[2]	748	PROsetta Stone, Neuro-QoL, NIH Toolbox	same as PHQ-9	Not mentioned.
BDI-II[2]	1,120	Op4G general population panel.	- 47.4% male<br>- 72.0% White<br>- Mean age: 46.4<br>- 58.1% with post-high school education.	28% scored ≥20 (moderate depression).
HADS Depression[2]	1,120	Op4G general population panel.	same as BDI-II	Not mentioned.
EPDS[3]	1,561	ECHO-PASS trial focusing on Prenatal exposure to alcohol.	- 11.6% American Indian/Alaska Native<br>- 86.7% White<br>- Mean age: 35<br>- 77% with post-high school education	Not mentioned.

References:

1. Pilkonis PA, Choi SW, Reise SP, Stover AM, Riley WT, Cella D. Item Banks for Measuring Emotional Distress From the Patient-Reported Outcomes Measurement Information System (PROMIS®): Depression, Anxiety, and Anger. *Assessment*. 2011;18(3):263-283. doi:10.1177/1073191111411667
2. Choi SW, Schalet B, Cook KF, Cella D. Establishing a common metric for depressive symptoms: Linking the BDI-II, CES-D, and PHQ-9 to PROMIS Depression. *Psychol Assess*. 2014;26(2):513-527. doi:10.1037/a0035768
3. Blackwell CK, Tang X, Elliott AJ, et al. Developing a common metric for depression across adulthood: Linking PROMIS depression with the Edinburgh Postnatal Depression Scale. *Psychol Assess*. 2021;33(7):610-618. doi:10.1037/pas0001009

© 2025 Riazy L et al. *JAMA Network Open*.

eAppendix. Country-Specific Demographics Tables

This section shows demographic information of the population that provided the PHQ-8 replies. It is structures in subsections for each of the 29 European countries. All information on groups of people consisting of less than 20 or between 20 and 50 need to be denoted as belonging to this group instead of the actual number following the Eurostat data privacy guidelines.

Austria

	Overall (N=15253)
**Age**	
15-19	845 (5.5%)
20-24	890 (5.8%)
25-29	1,019 (6.7%)
30-34	1,009 (6.6%)
35-39	1,078 (7.1%)
40-44	1,085 (7.1%)
45-49	1,224 (8.0%)
50-54	1,460 (9.6%)
55-59	1,465 (9.6%)
60-64	1,328 (8.7%)
65-69	1,094 (7.2%)
70-74	925 (6.1%)
75+	1,831 (12.0%)
**Sex**	
Female	8,173 (53.6%)
Male	7,080 (46.4%)
**Urbanization**	
Densely-populated	2,633 (17.3%)
Intermediate-populated	5,027 (33.0%)
Thinly-populated	7,593 (49.8%)
**Income**	
Below 1st quintile	3,271 (21.4%)
Between 1st quintile and 2nd quintile	3,097 (20.3%)
Between 2nd quintile and 3rd quintile	3,649 (23.9%)
Between 3rd quintile and 4th quintile	2,950 (19.3%)

© 2025 Riazy L et al. *JAMA Network Open*.

	Overall (N=15253)
Between 4th quintile and 5th quintile	2,286 (15.0%)
**Job_status**	
Employed	7,909 (51.9%)
Unemployed	502 (3.3%)
Retired	4,839 (31.7%)
Unable to work due to long-standing health problems	87 (0.6%)
Student, pupil	933 (6.1%)
Fulfilling domestic tasks	503 (3.3%)
Compulsory military or civilian service	20-49 (0.2%)
Other	442 (2.9%)
Missing	<20
**Marital_Status**	
Never married and never been in a registered partnership	4,687 (30.7%)
Married or in a registered partnership	8,145 (53.4%)
Widowed or in registered partnership that ended with death of partner	1,161 (7.6%)
Divorced or in registered partnership that was legally dissolved	1,260 (8.3%)

© 2025 Riazy L et al. *JAMA Network Open*.

Belgium

	Overall (N=8761)
**Age**	
15-19	394 (4.5%)
20-24	400 (4.6%)
25-29	556 (6.3%)
30-34	632 (7.2%)
35-39	691 (7.9%)
40-44	769 (8.8%)
45-49	790 (9.0%)
50-54	826 (9.4%)
55-59	789 (9.0%)
60-64	766 (8.7%)
65-69	654 (7.5%)
70-74	560 (6.4%)
75+	934 (10.7%)
**Sex**	
Female	4,598 (52.5%)
Male	4,163 (47.5%)
**Urbanization**	
Densely-populated	4,072 (46.5%)
Intermediate-populated	3,369 (38.5%)
Thinly-populated	1,320 (15.1%)
**Income**	
Below 1st quintile	1,203 (13.7%)
Between 1st quintile and 2nd quintile	1,245 (14.2%)
Between 2nd quintile and 3rd quintile	1,527 (17.4%)
Between 3rd quintile and 4th quintile	1,880 (21.5%)
Between 4th quintile and 5th quintile	2,055 (23.5%)
Missing	851 (9.7%)
**Job_status**	
Employed	4,406 (50.3%)

© 2025 Riazy L et al. *JAMA Network Open*.

	Overall (N=8761)
Unemployed	358 (4.1%)
Retired	2,268 (25.9%)
Unable to work due to long-standing health problems	413 (4.7%)
Student, pupil	819 (9.3%)
Fulfilling domestic tasks	329 (3.8%)
Compulsory military or civilian service	<20
Other	<20
Missing	168 (1.9%)
**Marital_Status**	
Never married and never been in a registered partnership	2,404 (27.4%)
Married or in a registered partnership	4,792 (54.7%)
Widowed or in registered partnership that ended with death of partner	634 (7.2%)
Divorced or in registered partnership that was legally dissolved	931 (10.6%)

© 2025 Riazy L et al. *JAMA Network Open*.

Bulgaria

	Overall (N=7231)
**Age**	
15-19	299 (4.1%)
20-24	306 (4.2%)
25-29	338 (4.7%)
30-34	371 (5.1%)
35-39	433 (6.0%)
40-44	559 (7.7%)
45-49	663 (9.2%)
50-54	571 (7.9%)
55-59	575 (8.0%)
60-64	670 (9.3%)
65-69	693 (9.6%)
70-74	670 (9.3%)
75+	1,083 (15.0%)
**Sex**	
Female	3,930 (54.3%)
Male	3,301 (45.7%)
**Urbanization**	
Densely-populated	3,134 (43.3%)
Intermediate-populated	2,370 (32.8%)
Thinly-populated	1,727 (23.9%)
**Income**	
Below 1st quintile	1,110 (15.4%)
Between 1st quintile and 2nd quintile	1,265 (17.5%)
Between 2nd quintile and 3rd quintile	1,529 (21.1%)
Between 3rd quintile and 4th quintile	1,562 (21.6%)
Between 4th quintile and 5th quintile	1,504 (20.8%)
Missing	261 (3.6%)
**Job_status**	
Employed	3,273 (45.3%)

© 2025 Riazy L et al. *JAMA Network Open*.

	Overall (N=7231)
Unemployed	633 (8.8%)
Retired	2,635 (36.4%)
Unable to work due to long-standing health problems	147 (2.0%)
Student, pupil	380 (5.3%)
Fulfilling domestic tasks	148 (2.0%)
Compulsory military or civilian service	<20
Other	<20
Missing	<20
**Marital_Status**	
Never married and never been in a registered partnership	1,786 (24.7%)
Married or in a registered partnership	3,730 (51.6%)
Widowed or in registered partnership that ended with death of partner	1,222 (16.9%)
Divorced or in registered partnership that was legally dissolved	477 (6.6%)
Missing	<20

© 2025 Riazy L et al. *JAMA Network Open*.

Cyprus

	Overall (N=5906)
**Age**	
15-19	343 (5.8%)
20-24	346 (5.9%)
25-29	382 (6.5%)
30-34	416 (7.0%)
35-39	460 (7.8%)
40-44	411 (7.0%)
45-49	484 (8.2%)
50-54	485 (8.2%)
55-59	547 (9.3%)
60-64	495 (8.4%)
65-69	478 (8.1%)
70-74	408 (6.9%)
75+	651 (11.0%)
**Sex**	
Female	3,122 (52.9%)
Male	2,784 (47.1%)
**Urbanization**	
Densely-populated	3,417 (57.9%)
Intermediate-populated	1,123 (19.0%)
Thinly-populated	1,366 (23.1%)
**Income**	
Below 1st quintile	1,170 (19.8%)
Between 1st quintile and 2nd quintile	1,155 (19.6%)
Between 2nd quintile and 3rd quintile	1,136 (19.2%)
Between 3rd quintile and 4th quintile	1,128 (19.1%)
Between 4th quintile and 5th quintile	1,148 (19.4%)
Missing	169 (2.9%)
**Job_status**	
Employed	3,057 (51.8%)

© 2025 Riazy L et al. *JAMA Network Open*.

	Overall (N=5906)
Unemployed	279 (4.7%)
Retired	1,572 (26.6%)
Unable to work due to long-standing health problems	80 (1.4%)
Student, pupil	479 (8.1%)
Fulfilling domestic tasks	395 (6.7%)
Compulsory military or civilian service	20-49
Other	<20
Missing	<20
**Marital_Status**	
Never married and never been in a registered partnership	1,475 (25.0%)
Married or in a registered partnership	3,628 (61.4%)
Widowed or in registered partnership that ended with death of partner	452 (7.7%)
Divorced or in registered partnership that was legally dissolved	351 (5.9%)

© 2025 Riazy L et al. *JAMA Network Open*.

Czechia

	Overall (N=7884)
**Age**	
15-19	321 (4.1%)
20-24	350 (4.4%)
25-29	371 (4.7%)
30-34	457 (5.8%)
35-39	455 (5.8%)
40-44	585 (7.4%)
45-49	615 (7.8%)
50-54	551 (7.0%)
55-59	579 (7.3%)
60-64	689 (8.7%)
65-69	856 (10.9%)
70-74	851 (10.8%)
75+	1,204 (15.3%)
**Sex**	
Female	4,464 (56.6%)
Male	3,420 (43.4%)
**Urbanization**	
Densely-populated	1,954 (24.8%)
Intermediate-populated	2,854 (36.2%)
Thinly-populated	3,076 (39.0%)
**Income**	
Below 1st quintile	2,021 (25.6%)
Between 1st quintile and 2nd quintile	1,648 (20.9%)
Between 2nd quintile and 3rd quintile	1,396 (17.7%)
Between 3rd quintile and 4th quintile	1,367 (17.3%)
Between 4th quintile and 5th quintile	1,262 (16.0%)
Missing	190 (2.4%)
**Job_status**	
Employed	3,636 (46.1%)

© 2025 Riazy L et al. *JAMA Network Open*.

	Overall (N=7884)
Unemployed	121 (1.5%)
Retired	3,171 (40.2%)
Unable to work due to long-standing health problems	199 (2.5%)
Student, pupil	464 (5.9%)
Fulfilling domestic tasks	287 (3.6%)
Compulsory military or civilian service	<20
Other	<20
Missing	<20
**Marital_Status**	
Never married and never been in a registered partnership	1,819 (23.1%)
Married or in a registered partnership	3,631 (46.1%)
Widowed or in registered partnership that ended with death of partner	1,321 (16.8%)
Divorced or in registered partnership that was legally dissolved	1,113 (14.1%)

© 2025 Riazy L et al. *JAMA Network Open*.

Germany

	Overall (N=23001)
**Age**	
15-19	661 (2.9%)
20-24	806 (3.5%)
25-29	927 (4.0%)
30-34	1,120 (4.9%)
35-39	1,296 (5.6%)
40-44	1,353 (5.9%)
45-49	1,534 (6.7%)
50-54	2,319 (10.1%)
55-59	2,620 (11.4%)
60-64	2,508 (10.9%)
65-69	2,475 (10.8%)
70-74	1,833 (8.0%)
75+	3,549 (15.4%)
**Sex**	
Female	12,111 (52.7%)
Male	10,890 (47.3%)
**Urbanization**	
Densely-populated	9,681 (42.1%)
Intermediate-populated	8,714 (37.9%)
Missing	838 (3.6%)
Thinly-populated	3,768 (16.4%)
**Income**	
Below 1st quintile	2,743 (11.9%)
Between 1st quintile and 2nd quintile	3,647 (15.9%)
Between 2nd quintile and 3rd quintile	4,218 (18.3%)
Between 3rd quintile and 4th quintile	5,204 (22.6%)
Between 4th quintile and 5th quintile	6,732 (29.3%)
Missing	457 (2.0%)
**Job_status**	

© 2025 Riazy L et al. *JAMA Network Open*.

	Overall (N=23001)
Employed	12,221 (53.1%)
Unemployed	383 (1.7%)
Retired	7,990 (34.7%)
Unable to work due to long-standing health problems	511 (2.2%)
Student, pupil	1,055 (4.6%)
Fulfilling domestic tasks	527 (2.3%)
Compulsory military or civilian service	20-49
Other	246 (1.1%)
Missing	20-49
**Marital_Status**	
Never married and never been in a registered partnership	5,622 (24.4%)
Married or in a registered partnership	12,530 (54.5%)
Widowed or in registered partnership that ended with death of partner	2,593 (11.3%)
Divorced or in registered partnership that was legally dissolved	2,222 (9.7%)
Missing	20-49

© 2025 Riazy L et al. *JAMA Network Open*.

Denmark

	Overall (N=6629)
**Age**	
15-19	350 (5.3%)
20-24	316 (4.8%)
25-29	318 (4.8%)
30-34	327 (4.9%)
35-39	361 (5.4%)
40-44	417 (6.3%)
45-49	503 (7.6%)
50-54	615 (9.3%)
55-59	657 (9.9%)
60-64	622 (9.4%)
65-69	620 (9.4%)
70-74	653 (9.9%)
75+	870 (13.1%)
**Sex**	
Female	3,768 (56.8%)
Male	2,861 (43.2%)
**Urbanization**	
Densely-populated	2,333 (35.2%)
Intermediate-populated	1,365 (20.6%)
Thinly-populated	2,931 (44.2%)
**Income**	
Below 1st quintile	1,073 (16.2%)
Between 1st quintile and 2nd quintile	952 (14.4%)
Between 2nd quintile and 3rd quintile	914 (13.8%)
Between 3rd quintile and 4th quintile	1,204 (18.2%)
Between 4th quintile and 5th quintile	978 (14.8%)
Missing	1508 (22.7%)
**Job_status**	
Employed	2,986 (45.0%)

© 2025 Riazy L et al. *JAMA Network Open*.

	Overall (N=6629)
Unemployed	178 (2.7%)
Retired	2,244 (33.9%)
Unable to work due to long-standing health problems	20-49
Student, pupil	576 (8.7%)
Fulfilling domestic tasks	52 (0.8%)
Compulsory military or civilian service	<20
Other	212 (3.2%)
Missing	339 (5.1%)
**Marital_Status**	
Never married and never been in a registered partnership	1,579 (23.8%)
Married or in a registered partnership	3,837 (57.9%)
Widowed or in registered partnership that ended with death of partner	416 (6.3%)
Divorced or in registered partnership that was legally dissolved	797 (12.0%)

© 2025 Riazy L et al. *JAMA Network Open*.

Estonia

	Overall (N=4851)
**Age**	
15-19	243 (5.0%)
20-24	223 (4.6%)
25-29	289 (6.0%)
30-34	352 (7.3%)
35-39	356 (7.3%)
40-44	368 (7.6%)
45-49	366 (7.5%)
50-54	369 (7.6%)
55-59	425 (8.8%)
60-64	427 (8.8%)
65-69	423 (8.7%)
70-74	336 (6.9%)
75+	674 (13.9%)
**Sex**	
Female	2,838 (58.5%)
Male	2,013 (41.5%)
**Urbanization**	
Densely-populated	2,575 (53.1%)
Intermediate-populated	407 (8.4%)
Thinly-populated	1,869 (38.5%)
**Income**	
Below 1st quintile	1,001 (20.6%)
Between 1st quintile and 2nd quintile	1,006 (20.7%)
Between 2nd quintile and 3rd quintile	924 (19.0%)
Between 3rd quintile and 4th quintile	954 (19.7%)
Between 4th quintile and 5th quintile	966 (19.9%)
**Job_status**	
Employed	2,710 (55.9%)
Unemployed	169 (3.5%)

© 2025 Riazy L et al. *JAMA Network Open*.

	Overall (N=4851)
Retired	1,375 (28.3%)
Unable to work due to long-standing health problems	158 (3.3%)
Student, pupil	266 (5.5%)
Fulfilling domestic tasks	130 (2.7%)
Compulsory military or civilian service	<20
Other	20-49
Missing	<20
**Marital_Status**	
Never married and never been in a registered partnership	1,722 (35.5%)
Married or in a registered partnership	1,886 (38.9%)
Widowed or in registered partnership that ended with death of partner	603 (12.4%)
Divorced or in registered partnership that was legally dissolved	639 (13.2%)
Missing	<20

© 2025 Riazy L et al. *JAMA Network Open*.

Greece

	Overall (N=7848)
**Age**	
15-19	273 (3.5%)
20-24	348 (4.4%)
25-29	257 (3.3%)
30-34	353 (4.5%)
35-39	543 (6.9%)
40-44	618 (7.9%)
45-49	589 (7.5%)
50-54	657 (8.4%)
55-59	619 (7.9%)
60-64	664 (8.5%)
65-69	717 (9.1%)
70-74	712 (9.1%)
75+	1,498 (19.1%)
**Sex**	
Female	4,152 (52.9%)
Male	3,696 (47.1%)
**Urbanization**	
Densely-populated	3,885 (49.5%)
Intermediate-populated	1,662 (21.2%)
Thinly-populated	2,301 (29.3%)
**Income**	
Below 1st quintile	759 (9.7%)
Between 1st quintile and 2nd quintile	2,137 (27.2%)
Between 2nd quintile and 3rd quintile	2,129 (27.1%)
Between 3rd quintile and 4th quintile	2,254 (28.7%)
Between 4th quintile and 5th quintile	569 (7.3%)
**Job_status**	
Employed	2,824 (36.0%)
Unemployed	753 (9.6%)

© 2025 Riazy L et al. *JAMA Network Open*.

	Overall (N=7848)
Retired	2,834 (36.1%)
Unable to work due to long-standing health problems	71 (0.9%)
Student, pupil	500 (6.4%)
Fulfilling domestic tasks	836 (10.7%)
Compulsory military or civilian service	<20
Other	20-49
Missing	<20
**Marital_Status**	
Never married and never been in a registered partnership	1,875 (23.9%)
Married or in a registered partnership	4,070 (51.9%)
Widowed or in registered partnership that ended with death of partner	1,363 (17.4%)
Divorced or in registered partnership that was legally dissolved	540 (6.9%)

© 2025 Riazy L et al. *JAMA Network Open*.

Finland

	Overall (N=5911)
**Age**	
15-19	266 (4.5%)
20-24	210 (3.6%)
25-29	283 (4.8%)
30-34	317 (5.4%)
35-39	344 (5.8%)
40-44	364 (6.2%)
45-49	361 (6.1%)
50-54	424 (7.2%)
55-59	548 (9.3%)
60-64	585 (9.9%)
65-69	693 (11.7%)
70-74	732 (12.4%)
75+	784 (13.3%)
**Sex**	
Female	3,443 (58.2%)
Male	2,468 (41.8%)
**Urbanization**	
Densely-populated	2,339 (39.6%)
Intermediate-populated	1,976 (33.4%)
Thinly-populated	1,596 (27.0%)
**Income**	
Below 1st quintile	1,015 (17.2%)
Between 1st quintile and 2nd quintile	1,034 (17.5%)
Between 2nd quintile and 3rd quintile	1,048 (17.7%)
Between 3rd quintile and 4th quintile	1,067 (18.1%)
Between 4th quintile and 5th quintile	1,083 (18.3%)
Missing	664 (11.2%)
**Job_status**	
Employed	2,553 (43.2%)

© 2025 Riazy L et al. *JAMA Network Open*.

	Overall<br>(N=5911)
Unemployed	236 (4.0%)
Retired	2,256 (38.2%)
Unable to work due to long-standing health problems	158 (2.7%)
Student, pupil	471 (8.0%)
Fulfilling domestic tasks	86 (1.5%)
Compulsory military or civilian service	<20
Other	63 (1.1%)
Missing	79 (1.3%)
**Marital_Status**	
Never married and never been in a registered partnership	2,006 (33.9%)
Married or in a registered partnership	2,918 (49.4%)
Widowed or in registered partnership that ended with death of partner	432 (7.3%)
Divorced or in registered partnership that was legally dissolved	493 (8.3%)
Missing	62 (1.0%)

© 2025 Riazy L et al. *JAMA Network Open*.

France

	Overall (N=13908)
**Age**	
15-19	1,037 (7.5%)
20-24	793 (5.7%)
25-29	793 (5.7%)
30-34	965 (6.9%)
35-39	1,107 (8.0%)
40-44	1,038 (7.5%)
45-49	1,216 (8.7%)
50-54	1,205 (8.7%)
55-59	1,207 (8.7%)
60-64	1,236 (8.9%)
65-69	1,141 (8.2%)
70-74	927 (6.7%)
75+	1,243 (8.9%)
**Sex**	
Female	7,363 (52.9%)
Male	6,545 (47.1%)
**Urbanization**	
Densely-populated	5,159 (37.1%)
Intermediate-populated	3,610 (26.0%)
Thinly-populated	5,139 (37.0%)
**Income**	
Below 1st quintile	2,751 (19.8%)
Between 1st quintile and 2nd quintile	2,772 (19.9%)
Between 2nd quintile and 3rd quintile	2,792 (20.1%)
Between 3rd quintile and 4th quintile	2,800 (20.1%)
Between 4th quintile and 5th quintile	2,793 (20.1%)
**Job_status**	
Employed	7,036 (50.6%)
Unemployed	798 (5.7%)

© 2025 Riazy L et al. *JAMA Network Open*.

	Overall (N=13908)
Retired	3,889 (28.0%)
Unable to work due to long-standing health problems	330 (2.4%)
Student, pupil	1,239 (8.9%)
Fulfilling domestic tasks	436 (3.1%)
Compulsory military or civilian service	<20
Other	178 (1.3%)
Missing	<20
**Marital_Status**	
Never married and never been in a registered partnership	4,593 (33.0%)
Married or in a registered partnership	7,217 (51.9%)
Widowed or in registered partnership that ended with death of partner	814 (5.9%)
Divorced or in registered partnership that was legally dissolved	1,220 (8.8%)
Missing	64 (0.5%)

© 2025 Riazy L et al. *JAMA Network Open*.

Croatia

	Overall (N=5230)
**Age**	
15-19	199 (3.8%)
20-24	220 (4.2%)
25-29	252 (4.8%)
30-34	291 (5.6%)
35-39	317 (6.1%)
40-44	309 (5.9%)
45-49	310 (5.9%)
50-54	445 (8.5%)
55-59	487 (9.3%)
60-64	567 (10.8%)
65-69	493 (9.4%)
70-74	387 (7.4%)
75+	953 (18.2%)
**Sex**	
Female	2,938 (56.2%)
Male	2,292 (43.8%)
**Urbanization**	
Densely-populated	1,005 (19.2%)
Intermediate-populated	1,914 (36.6%)
Thinly-populated	2,311 (44.2%)
**Income**	
Below 1st quintile	652 (12.5%)
Between 1st quintile and 2nd quintile	653 (12.5%)
Between 2nd quintile and 3rd quintile	645 (12.3%)
Between 3rd quintile and 4th quintile	641 (12.3%)
Between 4th quintile and 5th quintile	647 (12.4%)
Missing	1992 (38.1%)
**Job_status**	
Employed	1,904 (36.4%)

© 2025 Riazy L et al. *JAMA Network Open*.

	Overall<br>(N=5230)
Unemployed	550 (10.5%)
Retired	2,284 (43.7%)
Unable to work due to long-standing health problems	52 (1.0%)
Student, pupil	234 (4.5%)
Fulfilling domestic tasks	162 (3.1%)
Compulsory military or civilian service	<20
Other	20-49
Missing	<20
**Marital_Status**	
Never married and never been in a registered partnership	995 (19.0%)
Married or in a registered partnership	3,095 (59.2%)
Widowed or in registered partnership that ended with death of partner	852 (16.3%)
Divorced or in registered partnership that was legally dissolved	234 (4.5%)
Missing	54 (1.0%)

© 2025 Riazy L et al. *JAMA Network Open*.

Hungary

	Overall (N=5603)
**Age**	
15-19	301 (5.4%)
20-24	311 (5.6%)
25-29	312 (5.6%)
30-34	352 (6.3%)
35-39	379 (6.8%)
40-44	509 (9.1%)
45-49	474 (8.5%)
50-54	448 (8.0%)
55-59	389 (6.9%)
60-64	500 (8.9%)
65-69	552 (9.9%)
70-74	435 (7.8%)
75+	641 (11.4%)
**Sex**	
Female	3,031 (54.1%)
Male	2,572 (45.9%)
**Urbanization**	
Densely-populated	1,826 (32.6%)
Intermediate-populated	1,863 (33.3%)
Thinly-populated	1,914 (34.2%)
**Income**	
Below 1st quintile	1,153 (20.6%)
Between 1st quintile and 2nd quintile	1,173 (20.9%)
Between 2nd quintile and 3rd quintile	1,139 (20.3%)
Between 3rd quintile and 4th quintile	1,269 (22.6%)
Between 4th quintile and 5th quintile	869 (15.5%)
**Job_status**	
Employed	2,806 (50.1%)
Unemployed	190 (3.4%)

© 2025 Riazy L et al. *JAMA Network Open*.

	Overall (N=5603)
Retired	1,800 (32.1%)
Unable to work due to long-standing health problems	150 (2.7%)
Student, pupil	354 (6.3%)
Fulfilling domestic tasks	219 (3.9%)
Compulsory military or civilian service	<20
Other	84 (1.5%)
Missing	<20
**Marital_Status**	
Never married and never been in a registered partnership	1,522 (27.2%)
Married or in a registered partnership	2,777 (49.6%)
Widowed or in registered partnership that ended with death of partner	710 (12.7%)
Divorced or in registered partnership that was legally dissolved	534 (9.5%)
Missing	60 (1.1%)

© 2025 Riazy L et al. *JAMA Network Open*.

Ireland

	Overall (N=7621)
**Age**	
15-19	425 (5.6%)
20-24	271 (3.6%)
25-29	357 (4.7%)
30-34	555 (7.3%)
35-39	786 (10.3%)
40-44	756 (9.9%)
45-49	614 (8.1%)
50-54	440 (5.8%)
55-59	717 (9.4%)
60-64	689 (9.0%)
65-69	709 (9.3%)
70-74	558 (7.3%)
75+	744 (9.8%)
**Sex**	
Female	4,138 (54.3%)
Male	3,483 (45.7%)
**Urbanization**	
Densely-populated	2,499 (32.8%)
Intermediate-populated	1,416 (18.6%)
Thinly-populated	3,706 (48.6%)
**Income**	
Below 1st quintile	1,304 (17.1%)
Between 1st quintile and 2nd quintile	1,112 (14.6%)
Between 2nd quintile and 3rd quintile	717 (9.4%)
Between 3rd quintile and 4th quintile	639 (8.4%)
Between 4th quintile and 5th quintile	457 (6.0%)
Missing	3392 (44.5%)
**Job_status**	
Employed	3,867 (50.7%)

© 2025 Riazy L et al. *JAMA Network Open*.

	Overall (N=7621)
Unemployed	<20
Retired	237 (3.1%)
Unable to work due to long-standing health problems	443 (5.8%)
Student, pupil	1,833 (24.1%)
Fulfilling domestic tasks	388 (5.1%)
Compulsory military or civilian service	786 (10.3%)
Other	50 (0.7%)
Missing	<20
**Marital_Status**	
Never married and never been in a registered partnership	2,539 (33.3%)
Married or in a registered partnership	4,088 (53.6%)
Widowed or in registered partnership that ended with death of partner	738 (9.7%)
Divorced or in registered partnership that was legally dissolved	249 (3.3%)
Missing	<20

© 2025 Riazy L et al. *JAMA Network Open*.

Iceland

	Overall (N=3881)
**Age**	
15-19	280 (7.2%)
20-24	254 (6.5%)
25-29	285 (7.3%)
30-34	263 (6.8%)
35-39	268 (6.9%)
40-44	329 (8.5%)
45-49	316 (8.1%)
50-54	320 (8.2%)
55-59	352 (9.1%)
60-64	318 (8.2%)
65-69	295 (7.6%)
70-74	226 (5.8%)
75+	375 (9.7%)
**Sex**	
Female	2,043 (52.6%)
Male	1,838 (47.4%)
**Urbanization**	
Densely-populated	2,364 (60.9%)
Intermediate-populated	661 (17.0%)
Thinly-populated	856 (22.1%)
**Income**	
Below 1st quintile	767 (19.8%)
Between 1st quintile and 2nd quintile	767 (19.8%)
Between 2nd quintile and 3rd quintile	767 (19.8%)
Between 3rd quintile and 4th quintile	767 (19.8%)
Between 4th quintile and 5th quintile	766 (19.7%)
Missing	20-49
**Job_status**	
Employed	2,492 (64.2%)

© 2025 Riazy L et al. *JAMA Network Open*.

	Overall (N=3881)
Unemployed	68 (1.8%)
Retired	633 (16.3%)
Unable to work due to long-standing health problems	211 (5.4%)
Student, pupil	345 (8.9%)
Fulfilling domestic tasks	20-49
Compulsory military or civilian service	<20
Other	101 (2.6%)
Missing	<20
**Marital_Status**	
Never married and never been in a registered partnership	1,464 (37.7%)
Married or in a registered partnership	1,847 (47.6%)
Widowed or in registered partnership that ended with death of partner	177 (4.6%)
Divorced or in registered partnership that was legally dissolved	349 (9.0%)
Missing	20-49

© 2025 Riazy L et al. *JAMA Network Open*.

Italy

	Overall (N=39368)
**Age**	
15-19	1,782 (4.5%)
20-24	1,766 (4.5%)
25-29	1,770 (4.5%)
30-34	1,968 (5.0%)
35-39	2,315 (5.9%)
40-44	3,155 (8.0%)
45-49	3,683 (9.4%)
50-54	3,967 (10.1%)
55-59	3,684 (9.4%)
60-64	3,402 (8.6%)
65-69	3,223 (8.2%)
70-74	3,013 (7.7%)
75+	5,640 (14.3%)
**Sex**	
Female	21,041 (53.4%)
Male	18,327 (46.6%)
**Urbanization**	
Densely-populated	11,430 (29.0%)
Intermediate-populated	17,305 (44.0%)
Thinly-populated	10,633 (27.0%)
**Income**	
Below 1st quintile	6,903 (17.5%)
Between 1st quintile and 2nd quintile	7,697 (19.6%)
Between 2nd quintile and 3rd quintile	7,727 (19.6%)
Between 3rd quintile and 4th quintile	8,577 (21.8%)
Between 4th quintile and 5th quintile	8,464 (21.5%)
**Job_status**	
Employed	17,142 (43.5%)
Unemployed	3,163 (8.0%)

© 2025 Riazy L et al. *JAMA Network Open*.

	Overall (N=39368)
Retired	9,889 (25.1%)
Unable to work due to long-standing health problems	<20
Student, pupil	2,723 (6.9%)
Fulfilling domestic tasks	5,642 (14.3%)
Compulsory military or civilian service	<20
Other	805 (2.0%)
Missing	<20
**Marital_Status**	
Never married and never been in a registered partnership	11,184 (28.4%)
Married or in a registered partnership	22,275 (56.6%)
Widowed or in registered partnership that ended with death of partner	3,664 (9.3%)
Divorced or in registered partnership that was legally dissolved	2,243 (5.7%)
Missing	<20

© 2025 Riazy L et al. *JAMA Network Open*.

Lithuania

	Overall (N=4836)
**Age**	
15-19	266 (5.5%)
20-24	212 (4.4%)
25-29	282 (5.8%)
30-34	357 (7.4%)
35-39	302 (6.2%)
40-44	320 (6.6%)
45-49	379 (7.8%)
50-54	408 (8.4%)
55-59	463 (9.6%)
60-64	462 (9.6%)
65-69	356 (7.4%)
70-74	297 (6.1%)
75+	732 (15.1%)
**Sex**	
Female	2,860 (59.1%)
Male	1,976 (40.9%)
**Urbanization**	
Densely-populated	1,962 (40.6%)
Intermediate-populated	714 (14.8%)
Thinly-populated	2,160 (44.7%)
**Income**	
Below 1st quintile	799 (16.5%)
Between 1st quintile and 2nd quintile	1,016 (21.0%)
Between 2nd quintile and 3rd quintile	1,037 (21.4%)
Between 3rd quintile and 4th quintile	737 (15.2%)
Between 4th quintile and 5th quintile	628 (13.0%)
Missing	619 (12.8%)
**Job_status**	
Employed	2,509 (51.9%)

© 2025 Riazy L et al. *JAMA Network Open*.

	Overall<br>(N=4836)
Unemployed	284 (5.9%)
Retired	1,401 (29.0%)
Unable to work due to long-standing health problems	179 (3.7%)
Student, pupil	320 (6.6%)
Fulfilling domestic tasks	104 (2.2%)
Compulsory military or civilian service	<20
Other	20-49
Missing	<20
**Marital_Status**	
Never married and never been in a registered partnership	1,121 (23.2%)
Married or in a registered partnership	2,362 (48.8%)
Widowed or in registered partnership that ended with death of partner	639 (13.2%)
Divorced or in registered partnership that was legally dissolved	714 (14.8%)

© 2025 Riazy L et al. *JAMA Network Open*.

Luxembourg

	Overall (N=4504)
**Age**	
15-19	224 (5.0%)
20-24	262 (5.8%)
25-29	285 (6.3%)
30-34	370 (8.2%)
35-39	449 (10.0%)
40-44	421 (9.3%)
45-49	457 (10.1%)
50-54	498 (11.1%)
55-59	421 (9.3%)
60-64	352 (7.8%)
65-69	315 (7.0%)
70-74	223 (5.0%)
75+	227 (5.0%)
**Sex**	
Female	2,428 (53.9%)
Male	2,076 (46.1%)
**Urbanization**	
Densely-populated	864 (19.2%)
Intermediate-populated	2,032 (45.1%)
Thinly-populated	1,608 (35.7%)
**Income**	
Below 1st quintile	685 (15.2%)
Between 1st quintile and 2nd quintile	804 (17.9%)
Between 2nd quintile and 3rd quintile	681 (15.1%)
Between 3rd quintile and 4th quintile	756 (16.8%)
Between 4th quintile and 5th quintile	481 (10.7%)
Missing	1097 (24.4%)
**Job_status**	
Employed	2,484 (55.2%)

© 2025 Riazy L et al. *JAMA Network Open*.

	Overall (N=4504)
Unemployed	128 (2.8%)
Retired	992 (22.0%)
Unable to work due to long-standing health problems	90 (2.0%)
Student, pupil	400 (8.9%)
Fulfilling domestic tasks	159 (3.5%)
Compulsory military or civilian service	<20
Other	157 (3.5%)
Missing	83 (1.8%)
**Marital_Status**	
Never married and never been in a registered partnership	1,131 (25.1%)
Married or in a registered partnership	2,611 (58.0%)
Widowed or in registered partnership that ended with death of partner	159 (3.5%)
Divorced or in registered partnership that was legally dissolved	415 (9.2%)
Missing	188 (4.2%)

© 2025 Riazy L et al. *JAMA Network Open*.

Latvia

	Overall (N=5851)
**Age**	
15-19	446 (7.6%)
20-24	290 (5.0%)
25-29	373 (6.4%)
30-34	535 (9.1%)
35-39	452 (7.7%)
40-44	446 (7.6%)
45-49	443 (7.6%)
50-54	421 (7.2%)
55-59	520 (8.9%)
60-64	500 (8.5%)
65-69	415 (7.1%)
70-74	295 (5.0%)
75+	715 (12.2%)
**Sex**	
Female	3,421 (58.5%)
Male	2,430 (41.5%)
**Urbanization**	
Densely-populated	2,251 (38.5%)
Intermediate-populated	1,219 (20.8%)
Thinly-populated	2,381 (40.7%)
**Income**	
Below 1st quintile	1,200 (20.5%)
Between 1st quintile and 2nd quintile	1,299 (22.2%)
Between 2nd quintile and 3rd quintile	1,387 (23.7%)
Between 3rd quintile and 4th quintile	912 (15.6%)
Between 4th quintile and 5th quintile	1,053 (18.0%)
**Job_status**	
Employed	2,958 (50.6%)
Unemployed	457 (7.8%)

© 2025 Riazy L et al. *JAMA Network Open*.

	Overall (N=5851)
Retired	1,450 (24.8%)
Unable to work due to long-standing health problems	199 (3.4%)
Student, pupil	491 (8.4%)
Fulfilling domestic tasks	209 (3.6%)
Compulsory military or civilian service	<20
Other	75 (1.3%)
Missing	<20
**Marital_Status**	
Never married and never been in a registered partnership	1,794 (30.7%)
Married or in a registered partnership	2,614 (44.7%)
Widowed or in registered partnership that ended with death of partner	579 (9.9%)
Divorced or in registered partnership that was legally dissolved	864 (14.8%)

© 2025 Riazy L et al. *JAMA Network Open*.

Malta

	Overall (N=4356)
**Age**	
15-19	206 (4.7%)
20-24	269 (6.2%)
25-29	299 (6.9%)
30-34	300 (6.9%)
35-39	359 (8.2%)
40-44	363 (8.3%)
45-49	310 (7.1%)
50-54	292 (6.7%)
55-59	377 (8.7%)
60-64	404 (9.3%)
65-69	361 (8.3%)
70-74	393 (9.0%)
75+	423 (9.7%)
**Sex**	
Female	2,279 (52.3%)
Male	2,077 (47.7%)
**Urbanization**	
Densely-populated	1,961 (45.0%)
Intermediate-populated	2,243 (51.5%)
Thinly-populated	152 (3.5%)
**Income**	
Below 1st quintile	798 (18.3%)
Between 1st quintile and 2nd quintile	975 (22.4%)
Between 2nd quintile and 3rd quintile	820 (18.8%)
Between 3rd quintile and 4th quintile	886 (20.3%)
Between 4th quintile and 5th quintile	877 (20.1%)
**Job_status**	
Employed	2,247 (51.6%)
Unemployed	96 (2.2%)

© 2025 Riazy L et al. *JAMA Network Open*.

	Overall (N=4356)
Retired	1,063 (24.4%)
Unable to work due to long-standing health problems	<20
Student, pupil	<20
Fulfilling domestic tasks	<20
Compulsory military or civilian service	<20
Other	950 (21.8%)
Missing	<20
**Marital_Status**	
Never married and never been in a registered partnership	1,511 (34.7%)
Married or in a registered partnership	2,431 (55.8%)
Widowed or in registered partnership that ended with death of partner	333 (7.6%)
Divorced or in registered partnership that was legally dissolved	81 (1.9%)

© 2025 Riazy L et al. *JAMA Network Open*.

Netherlands

	Overall (N=8194)
**Age**	
15-19	574 (7.0%)
20-24	472 (5.8%)
25-29	560 (6.8%)
30-34	589 (7.2%)
35-39	562 (6.9%)
40-44	584 (7.1%)
45-49	620 (7.6%)
50-54	724 (8.8%)
55-59	768 (9.4%)
60-64	674 (8.2%)
65-69	629 (7.7%)
70-74	639 (7.8%)
75+	799 (9.8%)
**Sex**	
Female	4,187 (51.1%)
Male	4,007 (48.9%)
**Urbanization**	
Densely-populated	4,337 (52.9%)
Intermediate-populated	2,893 (35.3%)
Thinly-populated	964 (11.8%)
**Income**	
Below 1st quintile	1,267 (15.5%)
Between 1st quintile and 2nd quintile	1,169 (14.3%)
Between 2nd quintile and 3rd quintile	1,646 (20.1%)
Between 3rd quintile and 4th quintile	1,953 (23.8%)
Between 4th quintile and 5th quintile	2,091 (25.5%)
Missing	68 (0.8%)
**Job_status**	
Employed	4,374 (53.4%)

© 2025 Riazy L et al. *JAMA Network Open*.

	Overall (N=8194)
Unemployed	131 (1.6%)
Retired	1,539 (18.8%)
Unable to work due to long-standing health problems	287 (3.5%)
Student, pupil	795 (9.7%)
Fulfilling domestic tasks	583 (7.1%)
Compulsory military or civilian service	<20
Other	316 (3.9%)
Missing	169 (2.1%)
**Marital_Status**	
Never married and never been in a registered partnership	2,797 (34.1%)
Married or in a registered partnership	4,249 (51.9%)
Widowed or in registered partnership that ended with death of partner	470 (5.7%)
Divorced or in registered partnership that was legally dissolved	678 (8.3%)

© 2025 Riazy L et al. *JAMA Network Open*.

Norway

	Overall (N=7913)
**Age**	
15-19	471 (6.0%)
20-24	584 (7.4%)
25-29	557 (7.0%)
30-34	581 (7.3%)
35-39	610 (7.7%)
40-44	615 (7.8%)
45-49	683 (8.6%)
50-54	718 (9.1%)
55-59	660 (8.3%)
60-64	609 (7.7%)
65-69	606 (7.7%)
70-74	506 (6.4%)
75+	713 (9.0%)
**Sex**	
Female	3,940 (49.8%)
Male	3,973 (50.2%)
**Urbanization**	
Densely-populated	1,853 (23.4%)
Intermediate-populated	3,269 (41.3%)
Thinly-populated	2,791 (35.3%)
**Income**	
Below 1st quintile	1,446 (18.3%)
Between 1st quintile and 2nd quintile	1,555 (19.7%)
Between 2nd quintile and 3rd quintile	1,592 (20.1%)
Between 3rd quintile and 4th quintile	1,676 (21.2%)
Between 4th quintile and 5th quintile	1,637 (20.7%)
Missing	<20
**Job_status**	
Employed	4,794 (60.6%)

© 2025 Riazy L et al. *JAMA Network Open*.

	Overall<br>(N=7913)
Unemployed	156 (2.0%)
Retired	1,582 (20.0%)
Unable to work due to long-standing health problems	441 (5.6%)
Student, pupil	818 (10.3%)
Fulfilling domestic tasks	20-49
Compulsory military or civilian service	<20
Other	20-49
Missing	20-49
**Marital_Status**	
Never married and never been in a registered partnership	3,359 (42.4%)
Married or in a registered partnership	3,585 (45.3%)
Widowed or in registered partnership that ended with death of partner	392 (5.0%)
Divorced or in registered partnership that was legally dissolved	569 (7.2%)
Missing	<20

© 2025 Riazy L et al. *JAMA Network Open*.

Poland

	Overall (N=16865)
**Age**	
15-19	558 (3.3%)
20-24	671 (4.0%)
25-29	872 (5.2%)
30-34	1,054 (6.2%)
35-39	1,257 (7.5%)
40-44	1,352 (8.0%)
45-49	1,216 (7.2%)
50-54	1,217 (7.2%)
55-59	1,523 (9.0%)
60-64	1,938 (11.5%)
65-69	1,978 (11.7%)
70-74	1,370 (8.1%)
75+	1,859 (11.0%)
**Sex**	
Female	9,925 (58.8%)
Male	6,940 (41.2%)
**Urbanization**	
Densely-populated	4,885 (29.0%)
Intermediate-populated	3,861 (22.9%)
Thinly-populated	8,119 (48.1%)
**Income**	
Below 1st quintile	2,655 (15.7%)
Between 1st quintile and 2nd quintile	2,570 (15.2%)
Between 2nd quintile and 3rd quintile	2,557 (15.2%)
Between 3rd quintile and 4th quintile	2,432 (14.4%)
Between 4th quintile and 5th quintile	2,364 (14.0%)
Missing	4287 (25.4%)
**Job_status**	
Employed	7,532 (44.7%)

© 2025 Riazy L et al. *JAMA Network Open*.

	Overall (N=16865)
Unemployed	598 (3.5%)
Retired	6,139 (36.4%)
Unable to work due to long-standing health problems	569 (3.4%)
Student, pupil	764 (4.5%)
Fulfilling domestic tasks	820 (4.9%)
Compulsory military or civilian service	<20
Other	419 (2.5%)
Missing	<20
**Marital_Status**	
Never married and never been in a registered partnership	2,951 (17.5%)
Married or in a registered partnership	10,361 (61.4%)
Widowed or in registered partnership that ended with death of partner	2,440 (14.5%)
Divorced or in registered partnership that was legally dissolved	1,053 (6.2%)
Missing	60 (0.4%)

© 2025 Riazy L et al. *JAMA Network Open*.

Portugal

	Overall (N=14617)
**Age**	
15-19	505 (3.5%)
20-24	532 (3.6%)
25-29	502 (3.4%)
30-34	598 (4.1%)
35-39	863 (5.9%)
40-44	1,145 (7.8%)
45-49	1,150 (7.9%)
50-54	1,208 (8.3%)
55-59	1,351 (9.2%)
60-64	1,416 (9.7%)
65-69	1,385 (9.5%)
70-74	1,336 (9.1%)
75+	2,626 (18.0%)
**Sex**	
Female	8,295 (56.7%)
Male	6,322 (43.3%)
**Urbanization**	
Densely-populated	4,225 (28.9%)
Intermediate-populated	5,626 (38.5%)
Thinly-populated	4,766 (32.6%)
**Income**	
Below 1st quintile	2,808 (19.2%)
Between 1st quintile and 2nd quintile	3,575 (24.5%)
Between 2nd quintile and 3rd quintile	2,993 (20.5%)
Between 3rd quintile and 4th quintile	2,616 (17.9%)
Between 4th quintile and 5th quintile	2,625 (18.0%)
**Job_status**	
Employed	6,470 (44.3%)
Unemployed	1,004 (6.9%)

	Overall<br>(N=14617)
Retired	5,148 (35.2%)
Unable to work due to long-standing health problems	364 (2.5%)
Student, pupil	699 (4.8%)
Fulfilling domestic tasks	757 (5.2%)
Compulsory military or civilian service	<20
Other	141 (1.0%)
Missing	20-49
**Marital_Status**	
Never married and never been in a registered partnership	3,724 (25.5%)
Married or in a registered partnership	7,177 (49.1%)
Widowed or in registered partnership that ended with death of partner	2,231 (15.3%)
Divorced or in registered partnership that was legally dissolved	1,439 (9.8%)
Missing	20-49

© 2025 Riazy L et al. *JAMA Network Open*.

Romania

	Overall (N=15908)
**Age**	
15-19	874 (5.5%)
20-24	747 (4.7%)
25-29	733 (4.6%)
30-34	916 (5.8%)
35-39	1,105 (6.9%)
40-44	1,358 (8.5%)
45-49	1,494 (9.4%)
50-54	1,445 (9.1%)
55-59	1,040 (6.5%)
60-64	1,469 (9.2%)
65-69	1,683 (10.6%)
70-74	1,227 (7.7%)
75+	1,817 (11.4%)
**Sex**	
Female	8,335 (52.4%)
Male	7,573 (47.6%)
**Urbanization**	
Densely-populated	4,862 (30.6%)
Intermediate-populated	4,537 (28.5%)
Thinly-populated	6,509 (40.9%)
**Income**	
Below 1st quintile	2,929 (18.4%)
Between 1st quintile and 2nd quintile	3,009 (18.9%)
Between 2nd quintile and 3rd quintile	2,999 (18.9%)
Between 3rd quintile and 4th quintile	3,109 (19.5%)
Between 4th quintile and 5th quintile	3,060 (19.2%)
Missing	802 (5.0%)
**Job_status**	
Employed	7,257 (45.6%)

© 2025 Riazy L et al. *JAMA Network Open*.

	Overall (N=15908)
Unemployed	219 (1.4%)
Retired	5,723 (36.0%)
Unable to work due to long-standing health problems	184 (1.2%)
Student, pupil	1,086 (6.8%)
Fulfilling domestic tasks	1,295 (8.1%)
Compulsory military or civilian service	<20
Other	144 (0.9%)
Missing	<20
**Marital_Status**	
Never married and never been in a registered partnership	3,204 (20.1%)
Married or in a registered partnership	9,600 (60.3%)
Widowed or in registered partnership that ended with death of partner	2,191 (13.8%)
Divorced or in registered partnership that was legally dissolved	913 (5.7%)

© 2025 Riazy L et al. *JAMA Network Open*.

Serbia

	Overall (N=12463)
**Age**	
15-19	695 (5.6%)
20-24	685 (5.5%)
25-29	730 (5.9%)
30-34	773 (6.2%)
35-39	876 (7.0%)
40-44	976 (7.8%)
45-49	912 (7.3%)
50-54	983 (7.9%)
55-59	1,135 (9.1%)
60-64	1,171 (9.4%)
65-69	1,334 (10.7%)
70-74	907 (7.3%)
75+	1,286 (10.3%)
**Sex**	
Female	6,420 (51.5%)
Male	6,043 (48.5%)
**Urbanization**	
Missing	12,463 (100%)
**Income**	
Below 1st quintile	2,551 (20.5%)
Between 1st quintile and 2nd quintile	2,539 (20.4%)
Between 2nd quintile and 3rd quintile	2,543 (20.4%)
Between 3rd quintile and 4th quintile	2,503 (20.1%)
Between 4th quintile and 5th quintile	2,327 (18.7%)
**Job_status**	
Employed	4,455 (35.7%)
Unemployed	2,247 (18.0%)
Retired	3,997 (32.1%)
Unable to work due to long-standing health problems	55 (0.4%)

© 2025 Riazy L et al. *JAMA Network Open*.

	Overall (N=12463)
Student, pupil	923 (7.4%)
Fulfilling domestic tasks	639 (5.1%)
Compulsory military or civilian service	<20
Other	128 (1.0%)
Missing	<20
**Marital_Status**	
Never married and never been in a registered partnership	2,690 (21.6%)
Married or in a registered partnership	7,549 (60.6%)
Widowed or in registered partnership that ended with death of partner	1,571 (12.6%)
Divorced or in registered partnership that was legally dissolved	631 (5.1%)
Missing	20-49

© 2025 Riazy L et al. *JAMA Network Open*.

Sweden

	Overall (N=9757)
**Age**	
15-19	682 (7.0%)
20-24	545 (5.6%)
25-29	724 (7.4%)
30-34	872 (8.9%)
35-39	631 (6.5%)
40-44	723 (7.4%)
45-49	703 (7.2%)
50-54	790 (8.1%)
55-59	707 (7.2%)
60-64	719 (7.4%)
65-69	705 (7.2%)
70-74	730 (7.5%)
75+	1,226 (12.6%)
**Sex**	
Female	4,842 (49.6%)
Male	4,915 (50.4%)
**Urbanization**	
Densely-populated	4,160 (42.6%)
Intermediate-populated	3,817 (39.1%)
Missing	<20
Thinly-populated	1,765 (18.1%)
**Income**	
Below 1st quintile	1,249 (12.8%)
Between 1st quintile and 2nd quintile	1,586 (16.3%)
Between 2nd quintile and 3rd quintile	2,019 (20.7%)
Between 3rd quintile and 4th quintile	2,247 (23.0%)
Between 4th quintile and 5th quintile	2,586 (26.5%)
Missing	70 (0.7%)
**Job_status**	

© 2025 Riazy L et al. *JAMA Network Open*.

	Overall (N=9757)
Employed	5,299 (54.3%)
Unemployed	234 (2.4%)
Retired	2,050 (21.0%)
Unable to work due to long-standing health problems	250 (2.6%)
Student, pupil	856 (8.8%)
Fulfilling domestic tasks	288 (3.0%)
Compulsory military or civilian service	<20
Other	320 (3.3%)
Missing	460 (4.7%)
**Marital_Status**	
Never married and never been in a registered partnership	3,742 (38.4%)
Married or in a registered partnership	4,384 (44.9%)
Widowed or in registered partnership that ended with death of partner	506 (5.2%)
Divorced or in registered partnership that was legally dissolved	1,110 (11.4%)
Missing	<20

© 2025 Riazy L et al. *JAMA Network Open*.

Slovenia

	Overall (N=9763)
**Age**	
15-19	585 (6.0%)
20-24	545 (5.6%)
25-29	516 (5.3%)
30-34	593 (6.1%)
35-39	773 (7.9%)
40-44	813 (8.3%)
45-49	795 (8.1%)
50-54	894 (9.2%)
55-59	851 (8.7%)
60-64	906 (9.3%)
65-69	800 (8.2%)
70-74	614 (6.3%)
75+	1,078 (11.0%)
**Sex**	
Female	5,353 (54.8%)
Male	4,410 (45.2%)
**Urbanization**	
Densely-populated	1,601 (16.4%)
Intermediate-populated	3,478 (35.6%)
Thinly-populated	4,684 (48.0%)
**Income**	
Below 1st quintile	1,320 (13.5%)
Between 1st quintile and 2nd quintile	1,522 (15.6%)
Between 2nd quintile and 3rd quintile	2,494 (25.5%)
Between 3rd quintile and 4th quintile	2,003 (20.5%)
Between 4th quintile and 5th quintile	2,378 (24.4%)
Missing	20-49
**Job_status**	
Employed	4,824 (49.4%)

© 2025 Riazy L et al. *JAMA Network Open*.

	Overall (N=9763)
Unemployed	528 (5.4%)
Retired	3,236 (33.1%)
Unable to work due to long-standing health problems	75 (0.8%)
Student, pupil	910 (9.3%)
Fulfilling domestic tasks	99 (1.0%)
Compulsory military or civilian service	<20
Other	20-49
Missing	20-49
**Marital_Status**	
Never married and never been in a registered partnership	2,871 (29.4%)
Married or in a registered partnership	5,339 (54.7%)
Widowed or in registered partnership that ended with death of partner	875 (9.0%)
Divorced or in registered partnership that was legally dissolved	539 (5.5%)
Missing	139 (1.4%)

© 2025 Riazy L et al. *JAMA Network Open*.

Slovakia

	Overall (N=5527)
**Age**	
15-19	171 (3.1%)
20-24	250 (4.5%)
25-29	315 (5.7%)
30-34	344 (6.2%)
35-39	437 (7.9%)
40-44	464 (8.4%)
45-49	420 (7.6%)
50-54	495 (9.0%)
55-59	477 (8.6%)
60-64	653 (11.8%)
65-69	611 (11.1%)
70-74	434 (7.9%)
75+	456 (8.3%)
**Sex**	
Female	3,217 (58.2%)
Male	2,310 (41.8%)
**Urbanization**	
Densely-populated	1,240 (22.4%)
Intermediate-populated	1,900 (34.4%)
Thinly-populated	2,387 (43.2%)
**Income**	
Below 1st quintile	1,041 (18.8%)
Between 1st quintile and 2nd quintile	1,136 (20.6%)
Between 2nd quintile and 3rd quintile	1,118 (20.2%)
Between 3rd quintile and 4th quintile	1,112 (20.1%)
Between 4th quintile and 5th quintile	1,120 (20.3%)
**Job_status**	
Employed	2,591 (46.9%)
Unemployed	280 (5.1%)

© 2025 Riazy L et al. *JAMA Network Open*.

	Overall (N=5527)
Retired	1,888 (34.2%)
Unable to work due to long-standing health problems	231 (4.2%)
Student, pupil	284 (5.1%)
Fulfilling domestic tasks	67 (1.2%)
Compulsory military or civilian service	<20
Other	186 (3.4%)
Missing	<20
**Marital_Status**	
Never married and never been in a registered partnership	1,397 (25.3%)
Married or in a registered partnership	2,747 (49.7%)
Widowed or in registered partnership that ended with death of partner	811 (14.7%)
Divorced or in registered partnership that was legally dissolved	572 (10.3%)

© 2025 Riazy L et al. *JAMA Network Open*.

eAppendix 2. Model Selection for Quantile Regression

Introduction

In this study, quantile regression was used to describe the relationship between the outcome variable, the PROMIS depression score, and a set of confounders. The outcome variable, referred to as PV (plausible value), is an estimate of the underlying PROMIS depression score derived using imputation techniques. To account for the effects of confounders such as Age, Sex, and Country, a series of quantile regression models were tested and evaluated using the Bayesian Information Criterion (BIC).

Quantile Regression and Bayesian Information Criterion

Quantile regression is a technique that allows us to estimate the relationship between the outcome variable and confounders at different quantiles of the outcome.

The objective function (Koenker, 2005, Cambridge University Press) in quantile regression for the τ-th quantile is:

$$Q(\boldsymbol{\beta}_\tau) = \sum_{i:y_i \geq \mathbf{x}_i^T \boldsymbol{\beta}_\tau} \tau \, |y_i - \mathbf{x}_i^T \boldsymbol{\beta}_\tau| + \sum_{i:y_i < \mathbf{x}_i^T \boldsymbol{\beta}_\tau} (1-\tau) \, |y_i - \mathbf{x}_i^T \boldsymbol{\beta}_\tau|$$

A smaller value of $Q(\boldsymbol{\beta}_\tau)$ at the estimated coefficients $\widehat{\boldsymbol{\beta}}_\tau$ indicates a better fit.

Let $\hat{Q} = Q(\widehat{\boldsymbol{\beta}}_\tau)$ represent the minimized value of the Quantile Regression objective function (a sum of weighted errors).

The log-likelihood, $\ln(L)$, used in BIC can be approximated from $\hat{Q}$:

$$\ln(L) \approx \text{const}_1 - \text{const}_2 \cdot \hat{Q} \quad (\text{where } \text{const}_2 > 0),$$

Both const_1 and const_2 are computed from a parameter often called σ, which may be different between implementation but is fixed within our scope, since we only used the same function. Also, both constants depend on the quantile, which is why only within-quantile comparisons are permitted.

The standard BIC definition (Schwarz, 1978, Ann. Statist.) is:

$$\text{BIC} = \underbrace{k\ln(n)}_{\substack{\text{Complexity}\\ \text{Penalty}}} \underbrace{-2 * \ln(L)}_{\text{Fit Term}}$$

where k is the number of parameters and n is the sample size.

Substituting the approximation for $\ln(L)$, we get the BIC for Quantile Regression:

$$\text{BIC} \approx k\ln(n) - 2\left(\text{const}_1 - \text{const}_2 \cdot \hat{Q}\right)$$

Simplifying this leads to:

$$\text{BIC} \approx \underbrace{k\ln(n)}_{\substack{\text{Complexity}\\ \text{Penalty}}} + \underbrace{2*\text{const}_2 \cdot \hat{Q}}_{\substack{\text{Fit Term}\\ \text{(based on QR error)}}} + \text{const}_4$$

This implies that a lower BIC for equal complexity indicates a lower fit error.

Evaluated Models

The following models were fitted to describe the relationship between pv (the plausible value for the PROMIS Depression score) and confounders:

- pv ~ 1 (Intercept-only model)
- pv ~ Age
- pv ~ Sex
- pv ~ (Sex + Age)
- pv ~ Country
- pv ~ (Country + Age)
- pv ~ (Sex + Country)
- pv ~ (Sex + Country + Age)
- pv ~ Country*Sex + ns(Age, df = 2)
- pv ~ (Sex + Country + ns(Age, df = 2))
- pv ~ Country*Sex*Age
- pv ~ Country*Sex*ns(Age, df = 2)
- pv ~ Sex + Country*ns(Age, df = 2)
- pv ~ Country*(Sex + ns(Age, df = 2))

Here, the term ns(Age, df = 2) represents a natural spline basis for Age with 2 degrees of freedom. Spline bases are used to allow for a flexible, non-linear relationship between age and the outcome variable while controlling for overfitting. Natural splines ensure that the model remains smooth at the boundaries of the age range.

© 2025 Riazy L et al. *JAMA Network Open*.

The interaction terms (*) between variables such as Country and Sex or Country and Age allow for the possibility that the relationship between these variables and the outcome (depression score) might vary depending on the levels of the other variables.

Model Selection and Results
After fitting each of the models listed above, we evaluated them using BIC to identify the model that provides the best trade-off between model complexity and predictive accuracy.

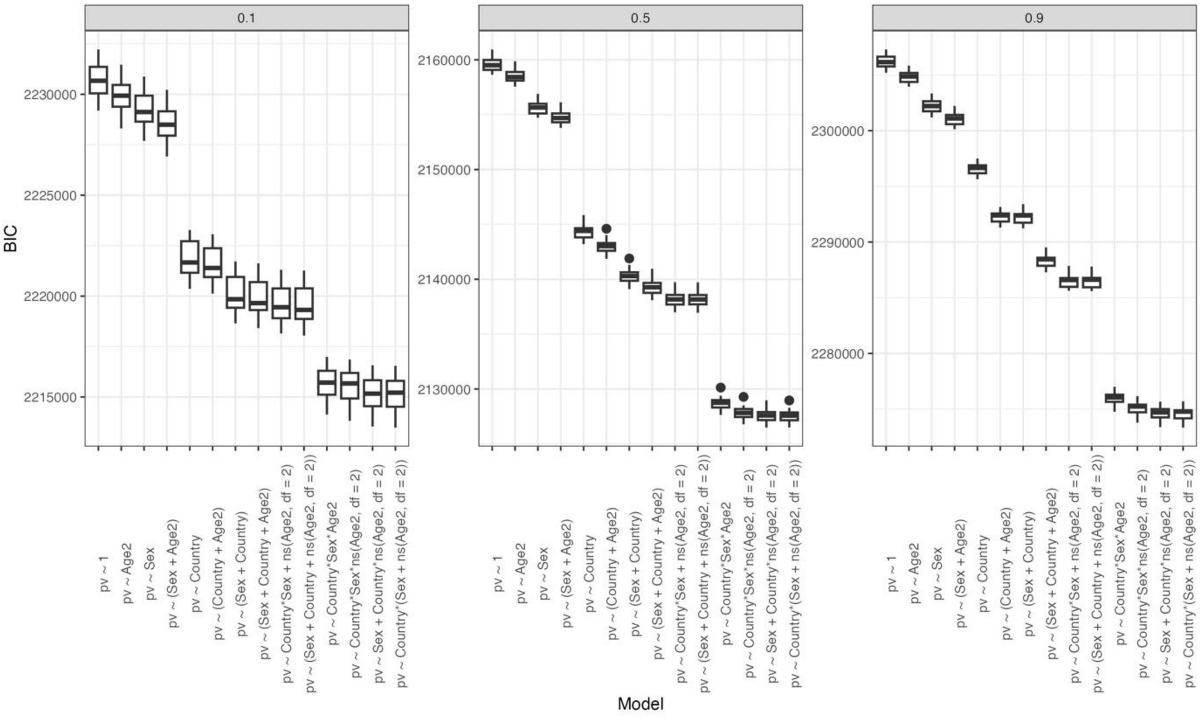

Figure 1: 3 boxplots for different quantile values (0.1, 0.5, 0.9). Each plot shows a collection of Bayesian Information Criterion values per formula. The BIC values were generated after fitting quantile regression models of the respective formula to each of 25 imputations of plausible PROMIS Depression Score values.

Figure 1 shows BIC values for different quantile values (0.1, 0.5, 0.9), as different quantiles may show different fitting behavior. Three visible clusters of models can be seen, where the lowest scoring, i.e. more favorable models are the following:

- Country*Sex*Age
- Country*Sex*ns(Age, df = 2)
- Sex + Country*ns(Age, df = 2)
- Country*(Sex + ns(Age, df = 2))

For a quantile value of tau = 0.5, the model with the lowest median BIC is Country*(Sex + ns(Age)). However most other models from within the lowest cluster have overlapping confidence interval regions.
The following table summarizes all models, which have an overlapping confidence interval with the model that showed the lowest median BIC.

Model	Median BIC	Lower CI	Upper CI	Overlap (%)
pv ~ Country*Sex*ns(Age, df = 2)	2,127,824	2,127,521	2,128,008	41.5%
pv ~ Sex + Country*ns(Age, df = 2)	2,127,592	2,127,249	2,127,740	96.5%
pv ~ Country*(Sex + ns(Age, df = 2))	2,127,575	2,127,232	2,127,723	100.0%

Since these models are not provably distinguishable from each other in terms of significant BIC difference, we chose the least computationally expensive model.

Final Model
Based on these considerations, the final selected model is:

Sex + Country*ns(Age, df = 2)

© 2025 Riazy L et al. *JAMA Network Open*.

This model allows us to capture the main effects of Sex and the interaction between Country and Age on the PROMIS depression score. It provides a well-balanced solution by maintaining sufficient model complexity to describe the relationships between the variables without overfitting.

© 2025 Riazy L et al. *JAMA Network Open*.

eTable 6. Parameters From Fitted Quantile Regression Model

In our study, we have fitted a quantile regression model (quantreg library in R). The following table shows all parameters necessary for reproducing the results we have presented.

The quantile is denoted as "tau" and model "Sex + Country*ns(Age, df = 2)" was used.

parameter	tau = 0.25	tau = 0.5	tau = 0.75	tau = 0.9	tau = 0.95	tau = 0.99
(Intercept)	34.50 (0.28)	39.23 (0.32)	43.85 (0.35)	48.06 (0.42)	50.74 (0.63)	57.60 (1.25)
SexMale	-1.84 (0.05)	-2.13 (0.06)	-2.20 (0.03)	-2.13 (0.06)	-2.08 (0.12)	-1.59 (0.21)
CountryCyprus	0.19 (1.13)	0.28 (1.07)	0.47 (1.15)	0.86 (1.53)	1.27 (2.06)	1.82 (4.58)
CountryGreece	0.27 (0.43)	0.38 (0.51)	0.60 (0.46)	0.95 (0.75)	1.27 (1.06)	0.81 (1.73)
CountryIreland	3.07 (0.53)	4.39 (0.49)	6.37 (0.52)	9.03 (0.58)	10.99 (0.90)	15.22 (2.22)
CountrySlovakia	1.14 (0.62)	1.32 (0.64)	1.49 (0.65)	1.80 (0.84)	2.19 (0.97)	3.92 (2.57)
CountryBulgaria	0.48 (0.55)	0.54 (0.50)	0.80 (0.50)	1.34 (0.63)	1.64 (0.86)	0.64 (2.08)
CountryItaly	2.50 (0.36)	3.68 (0.37)	5.38 (0.47)	6.71 (0.43)	7.29 (0.66)	7.91 (1.41)
CountryCzechia	1.69 (0.43)	2.34 (0.43)	3.44 (0.49)	4.49 (0.60)	4.92 (0.78)	5.03 (1.66)
CountryRomania	1.39 (0.37)	1.77 (0.40)	2.36 (0.44)	3.75 (0.46)	4.69 (0.70)	3.03 (1.38)
CountryLatvia	2.75 (0.68)	4.10 (0.74)	6.11 (0.75)	7.92 (1.05)	9.13 (1.40)	10.76 (3.04)
CountryNorway	7.96 (0.49)	10.02 (0.40)	11.60 (0.54)	12.95 (0.67)	13.88 (0.90)	14.22 (1.67)
CountryPoland	2.32 (0.39)	3.04 (0.44)	4.29 (0.48)	5.56 (0.52)	5.98 (0.75)	5.53 (1.87)
CountryAustria	6.46 (0.39)	8.14 (0.38)	9.15 (0.43)	9.66 (0.56)	9.85 (0.79)	9.25 (1.76)
CountryLithuania	3.58 (0.70)	5.11 (0.58)	7.69 (0.59)	9.91 (0.66)	11.15 (1.03)	12.15 (2.12)
CountryMalta	6.11 (1.61)	7.84 (1.53)	9.00 (1.67)	9.53 (2.18)	9.61 (3.07)	8.78 (6.67)
CountryFinland	9.79 (0.46)	12.39 (0.50)	14.58 (0.47)	17.25 (0.65)	18.73 (1.00)	18.11 (1.68)
CountryHungary	4.89 (0.48)	6.59 (0.47)	8.94 (0.51)	10.46 (0.57)	11.34 (1.01)	12.23 (1.65)
CountryCroatia	2.99 (0.55)	4.10 (0.50)	5.58 (0.63)	6.16 (0.72)	6.22 (1.01)	5.89 (2.24)
CountryGermany	9.78 (0.37)	11.30 (0.39)	12.07 (0.43)	12.80 (0.53)	13.42 (0.76)	12.85 (1.63)
CountryPortugal	4.06 (0.51)	5.57 (0.53)	7.14 (0.55)	8.45 (0.56)	9.14 (0.90)	9.64 (2.05)
CountryDenmark	11.76 (0.48)	14.02 (0.45)	15.67 (0.53)	17.69 (0.53)	20.16 (0.94)	21.14 (1.90)
CountrySweden	9.78 (0.50)	12.91 (0.43)	15.23 (0.47)	17.42 (0.60)	19.00 (0.67)	20.34 (1.47)
CountryBelgium	9.73 (0.58)	10.63 (0.49)	11.78 (0.50)	12.23 (0.68)	12.19 (0.71)	15.29 (2.38)
CountrySlovenia	9.33 (0.77)	11.84 (0.71)	13.37 (0.74)	14.45 (0.99)	15.41 (1.46)	16.48 (3.10)
CountryNetherlands	10.56 (0.40)	11.98 (0.37)	12.55 (0.41)	12.88 (0.45)	13.60 (0.67)	14.65 (1.75)
CountryFrance	10.39 (0.37)	11.93 (0.37)	12.85 (0.41)	13.33 (0.50)	13.60 (0.71)	12.70 (1.49)
CountryLuxembourg	10.64 (1.33)	13.21 (1.27)	15.02 (1.36)	16.97 (1.77)	18.29 (2.50)	20.08 (5.45)
CountryEstonia	9.68 (0.74)	11.33 (0.84)	12.44 (0.84)	13.27 (1.11)	13.92 (1.54)	14.47 (3.15)
CountryIceland	13.73 (1.85)	14.35 (1.77)	14.43 (1.94)	14.55 (2.51)	15.16 (3.54)	14.43 (7.65)
ns(Age2, df = 2)1	3.61 (0.63)	5.07 (0.65)	7.43 (0.76)	10.26 (0.92)	12.40 (1.40)	15.73 (2.71)
ns(Age2, df = 2)2	3.83 (0.35)	5.69 (0.33)	8.02 (0.39)	9.75 (0.50)	10.63 (0.69)	13.80 (1.36)

© 2025 Riazy L et al. *JAMA Network Open*.

CountryCyprus:ns(Age2, df = 2)1	-0.51 (2.72)	-0.20 (2.57)	0.48 (2.74)	0.78 (3.59)	0.24 (4.99)	-0.05 (11.06)
CountryGreece:ns(Age2, df = 2)1	-0.99 (1.01)	-1.20 (1.03)	-1.27 (1.02)	-1.70 (1.65)	-2.64 (2.12)	-1.65 (3.63)
CountryIreland:ns(Age2, df = 2)1	-4.53 (1.18)	-6.43 (1.13)	-8.97 (1.23)	-11.96 (1.28)	-14.31 (2.04)	-18.58 (4.96)
CountrySlovakia:ns(Age2, df = 2)1	1.60 (1.31)	2.16 (1.42)	2.51 (1.45)	2.25 (1.72)	1.32 (2.04)	-2.73 (5.55)
CountryBulgaria:ns(Age2, df = 2)1	1.31 (1.10)	2.13 (1.16)	2.60 (1.17)	2.93 (1.38)	3.08 (1.91)	6.93 (4.78)
CountryItaly:ns(Age2, df = 2)1	-0.90 (0.71)	-1.99 (0.74)	-4.54 (1.00)	-7.01 (0.95)	-8.16 (1.46)	-8.99 (2.95)
CountryCzechia:ns(Age2, df = 2)1	1.71 (1.08)	1.26 (0.97)	-0.77 (1.07)	-3.14 (1.30)	-4.55 (1.70)	-7.75 (3.25)
CountryRomania:ns(Age2, df = 2)1	3.28 (0.89)	4.98 (0.89)	5.43 (0.98)	2.71 (0.96)	-0.04 (1.47)	-0.05 (3.14)
CountryLatvia:ns(Age2, df = 2)1	1.62 (1.60)	0.43 (1.66)	-2.57 (1.72)	-6.23 (2.35)	-9.09 (3.16)	-13.57 (6.61)
CountryNorway:ns(Age2, df = 2)1	-9.59 (1.11)	-12.22 (0.89)	-14.34 (1.26)	-17.02 (1.52)	-19.12 (1.97)	-21.09 (3.96)
CountryPoland:ns(Age2, df = 2)1	1.89 (0.92)	2.22 (0.90)	0.76 (1.00)	-2.00 (1.14)	-3.30 (1.65)	-2.07 (3.94)
CountryAustria:ns(Age2, df = 2)1	-4.88 (0.80)	-6.66 (0.79)	-7.92 (1.02)	-8.94 (1.28)	-9.37 (1.81)	-9.92 (3.91)
CountryLithuania:ns(Age2, df = 2)1	-0.92 (1.61)	-1.63 (1.23)	-4.74 (1.32)	-8.92 (1.53)	-10.82 (2.27)	-9.96 (4.88)
CountryMalta:ns(Age2, df = 2)1	-4.37 (3.92)	-5.19 (3.75)	-6.55 (4.09)	-8.76 (5.34)	-10.26 (7.51)	-11.40 (16.32)
CountryFinland:ns(Age2, df = 2)1	-12.32 (1.07)	-15.17 (1.13)	-17.64 (1.04)	-22.83 (1.42)	-26.46 (1.95)	-25.08 (3.76)
CountryHungary:ns(Age2, df = 2)1	-1.46 (1.13)	-2.47 (0.95)	-5.80 (1.20)	-9.10 (1.20)	-11.72 (2.22)	-13.61 (3.53)
CountryCroatia:ns(Age2, df = 2)1	1.21 (1.24)	0.94 (1.10)	-0.40 (1.34)	0.04 (1.68)	0.48 (2.06)	0.76 (4.83)
CountryGermany:ns(Age2, df = 2)1	-9.00 (0.80)	-9.88 (0.80)	-10.25 (0.92)	-11.09 (1.21)	-11.36 (1.84)	-10.57 (3.49)
CountryPortugal:ns(Age2, df = 2)1	-0.68 (1.14)	-0.75 (1.15)	-1.29 (1.21)	-2.60 (1.34)	-2.72 (2.09)	-0.72 (4.32)
CountryDenmark:ns(Age2, df = 2)1	-14.98 (1.12)	-17.30 (1.00)	-18.63 (1.16)	-21.44 (1.04)	-24.88 (2.06)	-22.40 (4.14)
CountrySweden:ns(Age2, df = 2)1	-11.94 (1.00)	-15.02 (0.94)	-16.20 (1.05)	-17.99 (1.35)	-19.04 (1.57)	-20.11 (3.09)
CountryBelgium:ns(Age2, df = 2)1	-9.92 (1.21)	-9.71 (0.97)	-10.37 (1.10)	-9.40 (1.42)	-7.32 (1.70)	-13.12 (4.69)
CountrySlovenia:ns(Age2, df = 2)1	-8.95 (1.76)	-11.81 (1.64)	-13.23 (1.67)	-14.87 (2.19)	-16.59 (3.24)	-17.66 (6.92)
CountryNetherlands:ns(Age2, df = 2)1	-9.82 (0.93)	-10.57 (0.79)	-10.63 (0.93)	-10.38 (1.03)	-11.40 (1.53)	-13.29 (4.52)
CountryFrance:ns(Age2, df = 2)1	-8.32 (0.84)	-9.64 (0.79)	-10.69 (0.91)	-11.45 (1.07)	-11.62 (1.54)	-10.42 (3.39)
CountryLuxembourg:ns(Age2, df = 2)1	-9.71 (3.15)	-12.34 (3.01)	-14.38 (3.25)	-18.05 (4.22)	-19.98 (5.93)	-21.95 (13.24)
CountryEstonia:ns(Age2, df = 2)1	-5.16 (1.69)	-6.40 (1.84)	-8.00 (2.02)	-10.36 (2.59)	-12.41 (3.56)	-15.96 (7.28)
CountryIceland:ns(Age2, df = 2)1	-14.74 (4.18)	-14.23 (3.98)	-13.72 (4.38)	-13.91 (5.68)	-14.38 (8.05)	-11.76 (17.30)
CountryCyprus:ns(Age2, df = 2)2	-0.36 (1.81)	-0.07 (1.74)	0.14 (1.87)	-0.07 (2.45)	0.13 (3.44)	-0.32 (7.50)
CountryGreece:ns(Age2, df = 2)2	-1.32 (0.44)	-1.84 (0.43)	-2.21 (0.55)	-2.75 (0.57)	-2.94 (0.85)	-4.01 (1.69)
CountryIreland:ns(Age2, df =	-3.45 (0.61)	-5.23	-7.26	-9.40	-10.93	-16.42

© 2025 Riazy L et al. *JAMA Network Open*.

2)2		(0.58)	(0.66)	(0.70)	(1.03)	(2.50)
CountrySlovakia:ns(Age2, df = 2)2	1.87 (0.64)	1.42 (0.55)	0.36 (0.55)	-0.85 (0.72)	-1.30 (1.08)	-3.37 (1.98)
CountryBulgaria:ns(Age2, df = 2)2	2.78 (0.52)	3.06 (0.50)	2.72 (0.60)	3.09 (0.76)	3.73 (0.98)	5.04 (2.15)
CountryItaly:ns(Age2, df = 2)2	-0.36 (0.38)	-0.94 (0.35)	-2.39 (0.43)	-3.19 (0.55)	-2.96 (0.80)	-3.60 (1.43)
CountryCzechia:ns(Age2, df = 2)2	0.51 (0.44)	-0.28 (0.46)	-1.50 (0.54)	-2.72 (0.72)	-3.63 (0.97)	-6.81 (1.61)
CountryRomania:ns(Age2, df = 2)2	6.27 (0.44)	6.99 (0.40)	4.88 (0.52)	2.70 (0.66)	2.35 (0.79)	2.22 (1.57)
CountryLatvia:ns(Age2, df = 2)2	-0.63 (0.84)	-1.81 (0.75)	-3.94 (0.80)	-5.68 (1.09)	-6.82 (1.41)	-9.85 (3.07)
CountryNorway:ns(Age2, df = 2)2	-6.75 (0.61)	-9.35 (0.55)	-12.19 (0.57)	-14.94 (0.78)	-16.31 (1.00)	-20.60 (2.17)
CountryPoland:ns(Age2, df = 2)2	2.53 (0.38)	2.17 (0.44)	0.45 (0.47)	-1.03 (0.59)	-1.08 (0.80)	-3.29 (1.63)
CountryAustria:ns(Age2, df = 2)2	-2.24 (0.52)	-3.88 (0.48)	-5.79 (0.53)	-7.29 (0.66)	-8.27 (0.92)	-11.63 (1.69)
CountryLithuania:ns(Age2, df = 2)2	1.59 (0.69)	0.32 (0.65)	-2.61 (0.69)	-4.36 (0.87)	-4.69 (1.38)	-8.19 (2.82)
CountryMalta:ns(Age2, df = 2)2	-1.45 (2.39)	-2.87 (2.26)	-5.32 (2.47)	-7.70 (3.20)	-9.24 (4.57)	-14.75 (9.85)
CountryFinland:ns(Age2, df = 2)2	-7.92 (0.50)	-10.00 (0.57)	-11.90 (0.56)	-14.09 (0.81)	-15.71 (0.99)	-20.72 (1.98)
CountryHungary:ns(Age2, df = 2)2	-1.45 (0.61)	-2.19 (0.56)	-3.66 (0.53)	-4.96 (0.74)	-5.29 (1.05)	-4.78 (1.93)
CountryCroatia:ns(Age2, df = 2)2	2.68 (0.57)	1.87 (0.54)	0.21 (0.61)	-1.16 (0.83)	-1.79 (1.04)	-5.29 (1.90)
CountryGermany:ns(Age2, df = 2)2	-5.21 (0.44)	-7.59 (0.37)	-10.35 (0.47)	-12.95 (0.54)	-14.95 (0.83)	-18.37 (1.78)
CountryPortugal:ns(Age2, df = 2)2	-0.96 (0.52)	-1.47 (0.50)	-2.68 (0.48)	-3.01 (0.67)	-2.58 (0.95)	-6.79 (1.81)
CountryDenmark:ns(Age2, df = 2)2	-9.24 (0.56)	-11.82 (0.52)	-13.67 (0.49)	-14.75 (0.81)	-15.43 (1.25)	-15.86 (2.24)
CountrySweden:ns(Age2, df = 2)2	-7.70 (0.46)	-10.54 (0.40)	-12.39 (0.48)	-13.85 (0.60)	-15.13 (0.79)	-19.33 (1.76)
CountryBelgium:ns(Age2, df = 2)2	-7.35 (0.56)	-8.90 (0.46)	-11.06 (0.50)	-12.93 (0.78)	-14.45 (0.99)	-18.50 (1.88)
CountrySlovenia:ns(Age2, df = 2)2	-2.39 (0.87)	-3.86 (0.83)	-5.49 (0.92)	-6.33 (1.20)	-6.24 (1.69)	-8.37 (3.43)
CountryNetherlands:ns(Age2, df = 2)2	-6.62 (0.49)	-8.51 (0.40)	-10.45 (0.46)	-11.83 (0.66)	-12.94 (0.86)	-17.51 (2.02)
CountryFrance:ns(Age2, df = 2)2	-5.37 (0.40)	-6.81 (0.44)	-8.85 (0.49)	-9.99 (0.58)	-10.48 (0.79)	-14.45 (1.83)
CountryLuxembourg:ns(Age2, df = 2)2	-6.31 (2.05)	-8.42 (1.98)	-10.60 (2.13)	-12.23 (2.79)	-13.03 (3.95)	-13.95 (8.62)
CountryEstonia:ns(Age2, df = 2)2	-1.98 (0.99)	-4.18 (0.94)	-6.57 (1.04)	-8.47 (1.34)	-9.63 (1.86)	-13.03 (4.03)
CountryIceland:ns(Age2, df = 2)2	-8.36 (2.20)	-10.14 (2.10)	-12.38 (2.30)	-14.22 (2.96)	-15.29 (4.21)	-18.46 (9.07)

© 2025 Riazy L et al. *JAMA Network Open*.

eFigure 2. **PROMIS T-Score Distributions for 29 European Countries**

In the following plot, we show distributions from 29 European countries of the imputed PROMIS T-Score values. All densities were generated with the function geom_density of ggplot2 in R. Each plot shows two densities, the one from the female population in pink and the male population in blue. The countries are sorted alphabetically.

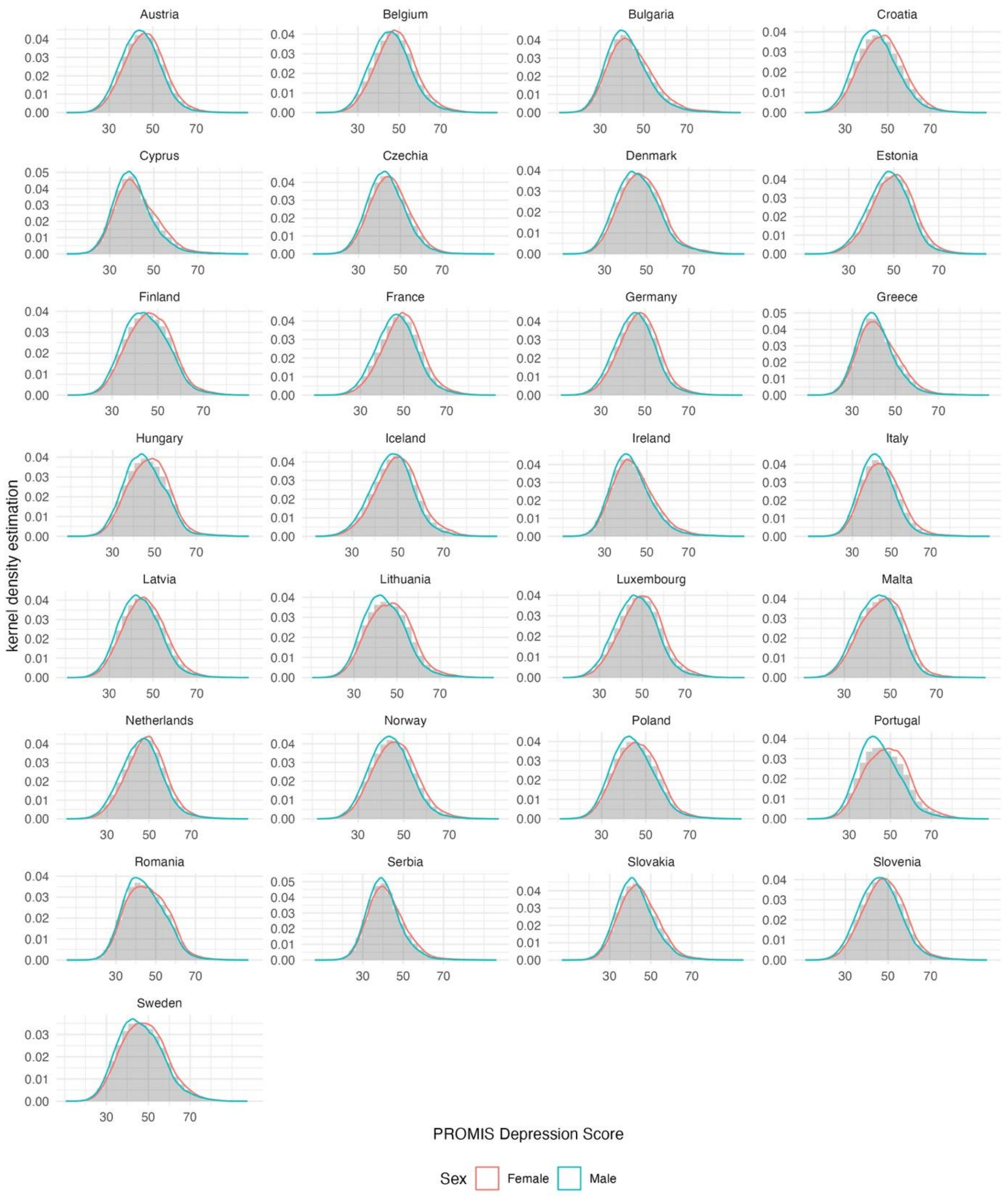

© 2025 Riazy L et al. *JAMA Network Open*.

eFigure 3. Quantile Regression Fit Quality Control

In our study, we have fitted a quantile regression model to imputed plausible values for PROMIS Depression T-Scores that were generated through item response theoretical linking from PHQ-8 responses.

The following plot shows an overlay of quantile regression fit evaluations versus the true quantile per age group, where all imputations have previously been combined into a single large set. The dots of the respective percentile color show the "true" value and the lines represent the fitted curve. The countries are sorted alphabetically.

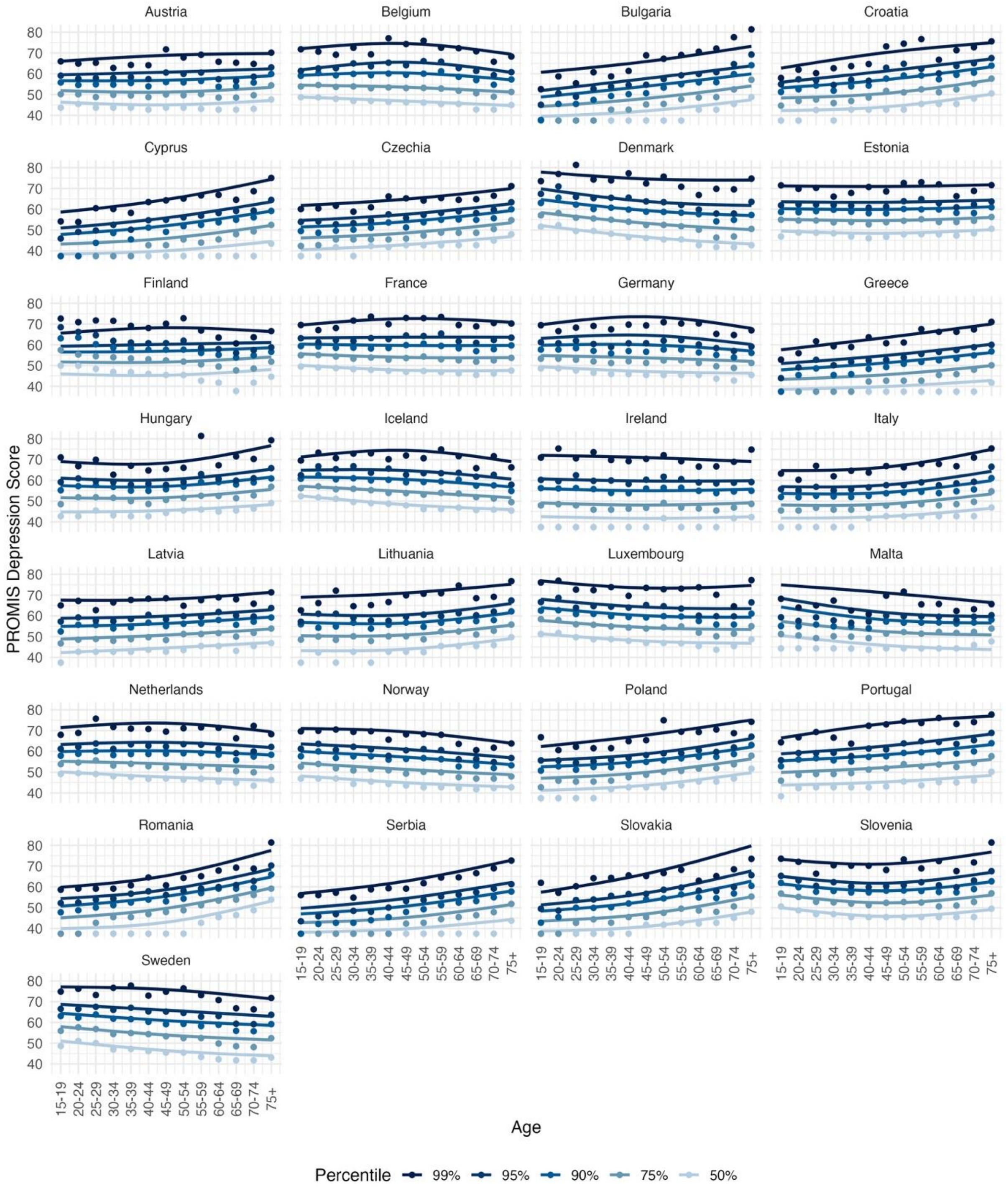

© 2025 Riazy L et al. *JAMA Network Open*.
