# Supplementary material for: Cross-Sectional Reference Data From 29 European Countries for 6 Frequently Used Depression Measures
Source: JAMA Netw Open. 2025 Jun 25;8(6):e2517394. doi: 10.1001/jamanetworkopen.2025.17394 (PMC12199077; doi:10.1001/jamanetworkopen.2025.17394)
Supplement: Supplement 2. — Data Sharing Statement [file jamanetwopen-e2517394-s002.pdf]

Data Sharing Statement

Riazy. Cross-Sectional Reference Data From 29 European Countries for 6 Frequently Used Depression Metrics. *JAMA Netw Open*. Published June 25, 2025. doi:10.1001/jamanetworkopen.2025.17394

Data
Data available: No

Additional Information
Explanation for why data not available: The data will be shared upon reasonable request by eurostat directly.
